# Tensor regularized total variation for denoising of third harmonic generation images of brain tumors

**DOI:** 10.1002/jbio.201800129

**Published:** 2018-08-16

**Authors:** Zhiqing Zhang, Marie L. Groot, Jan C. de Munck

**Affiliations:** ^1^ LaserLab Amsterdam, Department of Physics, Faculty of Sciences VU University Amsterdam The Netherlands; ^2^ Department of Radiology and Nuclear Medicine VU University Medical Center Amsterdam The Netherlands; ^3^ Amsterdam Neuroscience VU University Amsterdam The Netherlands

**Keywords:** anisotropic diffusion, convex optimization, tensor regularization, third harmonic generation, weak edges

## Abstract

Third harmonic generation (THG) microscopy shows great potential for instant pathology of brain tissue during surgery. However, the rich morphologies contained and the noise associated makes image restoration, necessary for quantification of the THG images, challenging. Anisotropic diffusion filtering (ADF) has been recently applied to restore THG images of normal brain, but ADF is hard‐to‐code, time‐consuming and only reconstructs salient edges. This work overcomes these drawbacks by expressing ADF as a tensor regularized total variation model, which uses the Huber penalty and the L_1_ norm for tensor regularization and fidelity measurement, respectively. The diffusion tensor is constructed from the structure tensor of ADF yet the tensor decomposition is performed only in the non‐flat areas. The resulting model is solved by an efficient and easy‐to‐code primal‐dual algorithm. Tests on THG brain tumor images show that the proposed model has comparable denoising performance as ADF while it much better restores weak edges and it is up to 60% more time efficient.

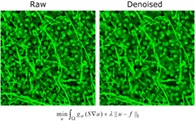

## Graphical abstract

Third harmonic generation (THG) microscopy is a label‐free imaging technique that shows great potential for in vivo instant pathology of human brain tissue. Here we have developed a robust and efficient image denoising method to facilitate the clinical applications of THG.

## 1 INTRODUCTION

Third harmonic generation (THG) microscopy 1, 2, 3 is a non‐linear imaging technique for label‐free three‐dimensional (3D) imaging of live tissues without the need for exogenous contrast agents. THG microscopy has established itself as an important tool for studying intact tissues such as insect embryos, plant seeds and intact mammalian tissue 2, epithelial tissues 4, 5, 6, zebrafish embryos 3, 7 and zebrafish nervous system 8. This technique has been applied for in vivo mouse brain imaging, revealing rich morphological information 9. Brain cells appear as dark holes on a bright background of neuropil, and axons and dendrites appear as bright fibers. More important, THG microscopy has shown great potential for clinical applications. Excellent agreement with the standard histopathology of skin cancers has been demonstrated for THG 10, 11 and THG also shows great potential for breast tumor diagnosis 12, 13.

In particular, we have recently demonstrated that THG yields high‐quality images of fresh, unstained human brain tumor tissue 14. Increased cellularity, nuclear pleomorphism, and rarefaction of neuropil have been clearly recognized in the acquired THG images of human brain tissue. This finding significantly facilitates the in vivo pathology of brain tumors and helps to reveal the tumor margins during surgery, which will improve the surgical outcomes.

Reliable image processing tools will strengthen the potential of THG microscopy for in vivo brain tumor pathology. In the image analysis pipeline of THG images of brain tissue, image denoising is essential and challenging due to the rich cellular morphologies and the low signal‐to‐noise ratio (SNR) 15. Anisotropic diffusion filtering (ADF) lies in the core of image denoising techniques that are able to remove strong image noise while maintaining the edges of objects sharp 16, 17, 18. The structure tensor is responsible for capturing the distribution of local gradients, thus enabling ADF to reconstruct certain kinds of structures, such as one‐dimensional (1D) flow‐like 17, 19, 20 and two‐dimensional (2D) membrane‐like structures 21, 22, as well as 2D blob and ridges 23. In a previous study 24, we have applied the classical edge‐enhancing ADF model 16 to restore the “dark” brain cells observed in THG images of mouse brain tissue. We have further developed in 15 a salient edge‐enhancing ADF model to reconstruct the rich morphologies appearing in THG images of structurally normal human brain tissue. However, all the existing ADF models have the drawback that the restored edges are in fact smooth 25. So far, most ADF models 19, 20, 21 are implemented using an explicit or semi‐implicit scheme 17, 26 to solve the diffusion equation which converges slowly.

The combination of ADF and the total variation (TV) model 27, 28, 29 provides an approach to overcome the drawbacks of the ADF models. TV regularization is another standard denoising method that has been studied mathematically for over decades 30, 31, 32, 33, 34, 35. In 25, an ADF model is formulated as a tensor regularized total variation (TRTV) model to restore the truly sharp edges, but the presented algorithm is based on gradient descent and has a slow convergence rate. The adaptive TRTV (ATRTV) model 36 improves convergence by using the primal‐dual algorithm 35 to solve the accompanying convex optimization model. The structure tensor adapts to the local geometry at each point but the estimated tensor may not reflect the true local structures if the image is corrupt by strong noise. Other important regularization approaches include the structure tensor total variation (STV) 37, 38 that penalizes the eigenvalues of the structure tensor, but STV does not make use of the directional information 36. The higher‐order regularizations such as the total generalized variation 39 and the Hessian Schatten‐Norm regularization (HS) 40 have also been proposed and also ignore direction of derivatives. There are many important alternative approaches to the image denoising problem such as dictionary learning based methods 41, sparse representation based methods 42, non‐local based methods 43, prior learning based methods 44, 45, 46, low‐rank based methods 47 and deep learning based methods 48.

In this study, we present a robust and efficient TRTV model that inherits the advantages of both ADF and TV, that is, their abilities of suppressing strong noise, estimating and restoring complex structures, and efficient convergence, to reconstruct 2D and 3D THG images of human brain tissue. The contributions of this study are 3‐fold. First, the pointwise decomposition of a structure tensor, which is time‐consuming and necessary for both ADF and TRTV, is greatly accelerated by performing the tensor decomposition only in the non‐flat areas. We use the gradient magnitude of a Gaussian at each point to estimate the first eigenvalue of the structure tensor and to distinguish flat from non‐flat areas. In the flat areas, the identity matrix is used as the diffusion tensor and no tensor decomposition is needed, while in the non‐flat regions, the tensor decomposition is applied to construct the application‐driven diffusion tensor. Second, existing TRTV models adopt the L_2_ norm for the data fidelity term while we use the L_1_ norm to make the proposed model (TRTV‐L_1_) robust to outliers and image contrast invariant. In previous work, it has been shown that geometrical features are better preserved by the TV models with the L_1_ norm 49. Third, we solve the TRTV‐L_1_ model with an efficient and easy‐to‐code primal‐dual algorithm as in 35, 36. In a detailed comparison of methods we show the ability of the TRTV‐L_1_ model to reconstruct weak edges, which is not well possible with other TRTV models. Weak edges are commonly observed in THG images and are important for clinical applications.

This work is a considerably extended version of the robust TRTV model previously presented at a conference 50. The rest of this paper is organized as follows: we review the existing TRTV models in Section 2. The proposed TRTV‐L_1_ model is explained in detail in Section 3. Simulated and real THG images are tested to demonstrate the efficiency and robustness of the proposed TRTV‐L_1_ model in Section 4. Conclusions follow in Section 5.

## 2 RELATED WORK

### 2.1 Anisotropic diffusion filtering and regularization

Let *u* denote an *m‐*dimensional (*m* = 2 or 3) image, and *f* be the noisy image. An ADF model 16, 17, 18, 19, 20, 21, 22, 23, 24, 51 has originally been defined by the partial differential equation (PDE) as follows:

(1) $$\partial_{t}u=\mathrm{div}\left(D\nabla u\right),u\left(x,t=0\right)=f,$$

together with an application‐driven diffusion tensor *D*, where the raw image *f* is used as the initial condition. *D* is computed from the gradient of a Gaussian smoothed version of the image *∇u*_*σ*_ in 3 consecutive steps. First, the structure tensor *J* is computed at each point to estimate the distribution of the local gradients:

(2) $$J_{\rho }\left(\nabla u_{\sigma }\right)={K_{\rho }}^{*}\left(\nabla u_{\sigma }⊗\nabla u_{\sigma }\right),\rho \geq 0.$$

Here *u*_*σ*_ is the Gaussian smoothed version of *u*, that is, *u* is convolved with a Gaussian kernel *K* of SD, *σ*,

(3) $$\left(K_{\sigma }*u\right)=\int_{{\mathbb{R}}^{m}}K_{\sigma }\left(x-y\right)u\left(y\right)dy,$$

(4) $$K_{\sigma }=\frac{1}{{\left(2{\pi \sigma }^{2}\right)}^{m/2}}\exp\left(-{\frac{|x|}{2\sigma^{2}}}^{2}\right).$$

The SD, *σ*, denotes the noise scale of the target image 17. To study the distribution of the local gradients, the outer product of *∇u*_*σ*_ is computed and each component of the resulting matrix is convolved with another Gaussian *K* of SD, *ρ*. *ρ* is the integration scale that reflects the characteristic size of the texture, and usually it is large in comparison to the noise scale *σ* 17.

Second, the structure tensor *J* is decomposed into the product of a diagonal matrix with eigenvalues *μ*_*i*_ and a matrix of eigenvectors **q**_*i*_ that indicate the distribution of the local gradients 17:

(5) $$J=Q\;\mathrm{diag}\left(\mu_{i}\right)Q^{T}.$$

The diagonal matrix, diag(*μ*_*i*_), is the eigenvalue matrix of all the eigenvalues ordered in the descending order, and the matrix *Q* is formed by the corresponding eigenvectors **q**_*i*_.

Finally, the eigenvalue values in (5) are replaced by the application‐driven diffusion matrix diag(*λ*_*i*_):

(6) $$D=Q\;\mathrm{diag}\left(\lambda_{i}\right)Q^{T},$$

where *λ*_*i*_ represents the amount of diffusivity along the eigenvector **q**_*i*_.

By taking the input image *f* as the starting point and evolving Eq. 1 over some time, the image is smoothed in flat areas and along the object edges, whereas the prominent edges themselves are maintained. Both the explicit and semi‐implicit schemes 17 have been widely employed to implement Eq. 1. The explicit scheme is easy‐to‐code yet converging slowly. The semi‐implicit scheme is more efficient because a larger time step is allowed, but harder to code because the inverse of a large matrix is involved.

Mathematically, Eq. 1 closely relates to the regularization problem that is designed to achieve a balance between smoothness and closeness to the input image *f*:

(7) $$\min_{u}R\left(u\right)+\lambda \|u-f\|.$$

In this functional, the first term is the regularization term (regularizer) that depends on the diffusion tensor *D.* The second term is the data fidelity term that uses a mathematical norm ||.|| to measure the closeness of *u* to the input image *f.* The implementation of this functional therefore depends on the construction of the diffusion tensor *D*, the choice of the regularizer and the fidelity norm. If we use the L_2_ norm for the data fidelity and substitute:

(8) $$R\left(u\right)=\int_{\Omega }\nabla u^{T}D\nabla u=\int_{\Omega }{\left|S\nabla u\right|}^{2},\;\text{with}\;D=S^{T}S,$$

into Eq. 7, its E‐L equation has the form:

(9) $$\partial_{t}u=\mathrm{div}\left(D\nabla u\right)-\lambda \left(u-f\right),$$

which has the same diffusion tensor as (1). Because *∇u* appears quadratically, *R* behaves as a L_2_ regularizer which has been shown unable to recover truly sharp edges 25, and the relation between (9) and (1) explains why the output of ADF is intrinsically smooth.

### 2.2 Total variation

Another standard image denoising method is the TV model that was introduced into compute vision first by Rudin, Osher and Fatemi (ROF) 27 as follows:

(10) $$\min_{u}\int_{\Omega }|\nabla u|+\lambda {\left\|u-f\right\|}_{2}^{2}.$$

The TV regularization penalizes only the total height of a slope but not its steepness, which permits the presence of edges in the minimizer. Although the ROF model permits prominent edges, it tends to create the so‐called *stair‐casing* effect and the primal minimization method used converges slowly. To address these drawbacks, several modifications have been made to reduce the *stair‐casing* effect and accelerate the convergence rate: replacing the TV regularization by Huber regularization, replacing the L_2_ norm by the L_1_ or Huber norm 52, and solving the convex minimization problem by the Chambolle's dual method 31, the split Bregman method 33, or the hybrid primal‐dual method 30, 32, 34, 35, 53. These first‐order primal‐dual algorithms enable easy‐to‐code implementation of the TV model. However, all these methods cannot properly remove the noise on the edges and cannot restore certain structures like 1D line‐like structures, because only the modulus of a gradient is considered in the regularizer, not its directions. Total variation based methods have also been applied to other image processing fields such as compressive sensing, mixed noise removal and image deblurring of natural and brain images 54, 55, 56.

### 2.3 Anisotropic total variation filtering

In order to overcome the problems of ADF and TV and combine their benefits, it is helpful to notice the close relation between diffusion filtering and regularization, which was initially studied in 57 for isotropic diffusion. The relation between anisotropic diffusion and the TV regularization was studied in 25, via the TRTV model as follows:

(11) $$\min_{u}\int_{\Omega }|S\nabla u|+\frac{\lambda }{2}{\left\|u-f\right\|}_{2}^{2}=\min_{u}\int_{\Omega }\sqrt{\nabla u^{T}D\nabla u}+\frac{\lambda }{2}{\left\|u-f\right\|}_{2}^{2}.$$

The matrix *S* satisfies *D* = *S*^*T*^*S*, with a given diffusion tensor *D.* The anisotropic regularizer used in (11) overcomes the drawbacks of ADF and reconstructs truly sharp edges. Because the directional information has been incorporated via the diffusion tensor *D* in this model, it is also able to remove the noise on the edges and restore the complex structure which is not possible with the TV model. Despite these improvements, the minimization used in 25 to solve this TRTV model was based on gradient descent which suffered from slow convergence.

The diffusion behavior of (11) can be analyzed in terms of the diffusion equation given by its E‐L equation:

(12) $$\partial_{t}u=\mathrm{div}\left(\frac{D}{|S\nabla u|}\nabla u\right)-\lambda \left(u-f\right).$$

The first term on the right corresponds to an ADF with the diffusion tensor *D*/ ∣ *S∇u*∣.

### 2.4 Adaptive regularization with the structure tensor

In 36, the convexity of the problem (11) was used to improve the computational performance of the TRTV model 25 by applying the primal‐dual algorithm 35, to solve the convex optimization of the proposed ATRTV model 36. Also, the Huber penalty *g*_*α*_ was used to regularize the structure tensor and reduce the *stair‐casing* effect caused by the TV regularization:

(13) $$\min_{u}\int_{\Omega }g_{\alpha }\left(S\nabla u\right)+\frac{\lambda }{2}{\left\|u-f\right\|}_{2}^{2},$$

(14) $$g_{\alpha }\left(\nabla u\right)=\left\{\begin{array}{cc} \frac{{\left|\nabla u\right|}^{2}}{2\alpha } & \text{if}\;|\nabla u|<\alpha ,\\ \frac{|\nabla u|-\alpha }{2} & \text{if}\;|\nabla u|\geq \alpha . \end{array}\right.$$

Here the matrix *S*, with $S=\max\left(\sqrt{\alpha },\sqrt[{4}]{\mu_{1}+\mu_{2}}\right)\;\mathrm{diag}{\left(\mu_{i}\right)}^{-1/2}Q^{T}$, is the adaptive tensor used to rotate and scale the axes of the local ellipse to coincide with the coordinate axes of the image domain. This design of the adaptive regularizer has taken into account the local structure of each point to penalize image variations. However, we noticed that the asymmetry of *S* may create artifacts and reduce the applicability of the algorithm in practice. We also note that the diffusion strength along the *i*th direction is approximately proportional to $1/\sqrt[{4}]{\mu_{i}}\ll 1$, which is not enough to suppress the noise when the input is corrupted by strong noise.

## 3 MATERIALS AND METHODS

### 3.1 Image samples and acquisition

All procedures on human tissue were performed with the approval of the Medical Ethical Committee of the VU University Medical Center and in accordance with Dutch license procedures and the declaration of Helsinki. All patients gave a written informed consent for tissue biopsy collection and signed a declaration permitting the use of their biopsy specimens in scientific research. We imaged brain tissue samples from 6 patients diagnosed with low‐grade glioma and 2 patients diagnosed with high‐grade glioma, as well as 2 structurally normal references with THG microscopy 14. Structurally normal brain samples were cut from the temporal cortex and subcortical white matter that had to be removed for the surgical treatment of deeper brain structures affected by epilepsy. Tumor brain samples were cut from tumor margin areas and from the tumor core and peritumoral areas. For details of the imaging setup, the tissue preparation and the tissue histology, we refer to previous works 9, 14.

### 3.2 The proposed tensor regularized total variation

When applied to THG images of brain tissue, all the methods above have their specific problems. The ADF models are computationally expensive and they cannot restore weak edges. The TV model creates the *stair‐casing* effect and cannot restore thin 1D line‐like structures. The existing TRTV models are either too expensive in computation or lack of enough denoising capability. To deal with these drawbacks and to make the TRTV approach applicable to THG images corrupted by strong noise, we present an efficient estimation of the diffusion tensor and we replace the L_2_ norm used in the data fidelity term by the robust L_1_ norm. We solve the resulting model by an efficient primal‐dual method.

#### 3.2.1 Efficient estimation of the diffusion tensor

One time‐consuming step of the ADF and TRTV models is that the diffusion matrix *D* or *S* needs to be estimated at each point to describe the distribution of local gradients. This is of no interest in flat areas because the gradients almost vanish. In 3D, this tensor decomposition procedure takes about half of the total computational time. If the tensor decomposition is only computed in non‐flat areas, the procedure will be substantially accelerated.

To do this, we exploit the fact that the flat regions consist of points whose first (largest) eigenvalue is small, and that this eigenvalue can be roughly estimated by |*∇u*_*σ*_|^2^ 16. This fact motivates the idea of thresholding |*∇u*_*σ*_|^2^ to distinguish flat and non‐flat regions. Before thresholding, we use the following function *g* to normalize and scale exponentially |*∇u*_*σ*_|^2^ to the range [0,1]:

(15) $$g\left(s\right)=\exp\left(\frac{-C_{4}}{{\left(s/\lambda \right)}^{4}}\right),\;s>0.$$

This function has been used in the edge‐enhancing ADF model 16 to define the diffusivity along the first direction. Following 16 we set *C*_4_ = 3.31488. *λ* is the threshold to control the trend of the function 16. Then we regard the points with *g*(|*∇u*_*σ*_|^2^) < *h* (here *h* is always set to 0.9) as the flat regions and the other points as the non‐flat regions. In the flat regions, the diffusion along each direction is isotropic and the diffusion tensor *D* reduces to the identity matrix *I.* In the non‐flat regions, the diffusion tensor *D* is defined as a weighted sum of the identity matrix and the application‐driven diffusion tensor, with the weight *g*(|*∇u*_*σ*_|^2^):

(16) $$\begin{array}{l} D=\left(1-g\left({\left|\nabla u_{\sigma }\right|}^{2}\right)\right)I+g\left({\left|\nabla u_{\sigma }\right|}^{2}\right)Q\;\mathrm{diag}\left(\lambda_{i}\right)Q^{T}\\ =Q\left(\left(1-g\left({\left|\nabla u_{\sigma }\right|}^{2}\right)\right)I+g\left({\left|\nabla u_{\sigma }\right|}^{2}\right)\;\mathrm{diag}\left(\lambda_{i}\right)\right)Q^{T}\\ ≔Q\Lambda Q^{T}. \end{array}$$

Therefore, the *g*(|*∇u*_*σ*_|^2^) has two roles here, one of which is acting as a threshold value and the other is acting as the weight for constructing the diffusion tensor *D* of the non‐flat areas. Note that most of the ADF and TRTV models could in principle be accelerated using the procedure described here with almost no loss of accuracy. When applied to 3D images, we use the following eigenvalue system to optimize the diffusivity*λ*_*i*_:

(17) $$\left\{\begin{array}{l} \lambda_{1}=1-g\left({\left|\nabla u_{\sigma }\right|}^{2}\right),\\ \lambda_{2}=\lambda_{1}-\left(\lambda_{1}-\lambda_{3}\right)h_{\tau }\left(C_{\text{plane}}\right),\\ \lambda_{3}=1. \end{array}\right.$$

For 2D THG images, the second diffusivity *λ*_2_ is ignored. *h*_*τ*_(⋅) is a fuzzy threshold function between 0 and 1 that allows a better control of the transition between 2D plane structures and other regions 21, 58, as follows:

(18) $$h_{\tau }\left(x\right)=\frac{\tanh\left[\gamma \left(x-\tau \right)\right]+1}{\tanh\left[\gamma \left(1.0-\tau \right)\right]+1},\;x\in \left[0,1\right].$$

where *γ* is a scaling factor that controls the transition and we set it to 100. *C*_plane_ is the plane‐confidence measure 21, 59 defined as follows:

(19) $$C_{\text{plane}}=\frac{\mu_{1}-\mu_{2}}{\mu_{1}+\mu_{2}}.$$

Smoothing behaviors of the diffusion matrix (17) are different for different regions: in background regions, *λ*_1_ is almost 1 and smoothing is encouraged from all the directions at an equal level (isotropic smoothing). In the vicinity of edges, *λ*_1_ ≈ 0, smoothing at the first direction is discouraged. In plane‐like regions, the fuzzy function *h*_*τ*_ tends to 1, and *λ*_2_ = 1, and smoothing at the second and third directions is allowed. In 1D structure regions, *λ*_2_ tends to *λ*_1_ and both are close to 0. Smoothing at the third direction is allowed only.

#### 3.2.2 Robust anisotropic regularization

Given a diffusion tensor *D* designed as (16), we consider the same regularizer as in Eq. 13 of the adaptive TRTV model 36:

(20) $$R\left(u\right)=\int_{\Omega }g_{\alpha }\left(S\nabla u\right),$$

but contrary to 36 we use a symmetric *S*, *S* = *D.* To analyze the behavior of this regularizer in terms of diffusion, we note that the E‐L equation that minimizes *R*(*u*) is:

(21) $$\partial_{t}u=\mathrm{div}\left(\frac{1}{\max\left(\alpha ,|S\nabla u|\right)}S^{T}S\nabla u\right).$$

To analyze the diffusion behavior along each eigenvector direction, we only need to estimate the ∣*S∇u*∣:

(22) $$\begin{array}{l} |S\nabla u|=|Q\Lambda Q^{T}\nabla u|=\sqrt{{\left(Q^{T}\nabla u\right)}^{T}\Lambda^{2}\left(Q^{T}\nabla u\right)}\\ =\sqrt{\sum_{i=1}^{3}\Lambda_{i}^{2}{\left(q_{i}^{T}\nabla u\right)}^{2}}\propto \sqrt{\sum_{i=1}^{3}\Lambda_{i}^{2}\mu_{i}}. \end{array}$$

Hence, the regularization problem (20) is a scaled version of the diffusion problem with the diffusion tensor *S*^*T*^*S* = *Q*Λ^2^*Q*^*T*^, whose behavior along each eigenvector is almost the same as the diffusion problem with diffusion tensor *D.* Note that in the flat regions, *S* becomes the identity matrix, and the regularization (20) reduced to the Huber regularization.

#### 3.2.3 Tensor regularized total variation‐L_1_

Different from the existing TRTV models, we consider the robust minimization problem as follows:

(23) $$\min_{u}\int_{\Omega }g_{\alpha }\left(S\nabla u\right)+\lambda {\left\|u-f\right\|}_{1},$$

where we have used the L_1_ norm in the data fidelity term. Compared to the L_2_ norm, the L_1_ norm is image contrast invariant, robust to noise and sensitive to fine details 49, 60.

#### 3.2.4 Numerical minimization

To efficiently solve the minimization problem (23), we note that it is a convex problem which can be reformulated as a saddle‐point problem. Therefore, it can be solved efficiently by the primal‐dual approach 34, 35, 36. To describe the problem in matrix algebra language, we reorder the image matrix *u* row‐wisely into a vector with *N* points, that is, *u* ∈ ℝ^*N*^. The minimization problem (23) is written as the following primal minimization problem:

(24) $$\min_{u}J\left(\mathrm{Au}\right)+\lambda {\left\|u-f\right\|}_{1},$$

where *Au*(*i*) = *S*(*i*)*∇u*(*i*) at each point *i*, and *J* denotes the Huber norm, $J\left(\mathrm{Au}\right)=\sum_{i=1}^{N}g_{\alpha }\left(\mathrm{Au}\left(i\right)\right)$.

To convert problem (24) into a primal‐dual problem, we introduce a dual variable *p* ∈ ℝ^*mN*^ (*m* = 2 or 3, the dimension of an image), and the convex conjugate of *J* (we refer to 61 for a complete introduction to the classical theory of convex analysis) is:

(25) $$J^{*}\left(p\right)=\underset{q\in {\mathbb{R}}^{\mathrm{mN}}}{\sup}〈p,q〉-J\left(q\right).$$

Since J^**^ = *J*, we have

(26) $$J\left(\mathrm{Au}\right)=\underset{p}{\sup}〈p,\mathrm{Au}〉-J^{*}\left(p\right).$$

Substituting (26) into (24), we obtain the equivalent saddle‐point problem of the minimization problem (24):

(27) $$\min_{u}\max_{p}〈p,\mathrm{Au}〉-J^{*}\left(p\right)+\lambda {\left\|u-f\right\|}_{1}.$$

According to the hybrid primal‐dual algorithm described in 34, 35, we need to solve the following dual, primal and approximate extra‐gradient steps iteratively,

(28) $$p^{k+1}=\underset{p}{\arg\max}〈p,A{\bar{u}}^{k}〉-J^{*}\left(p\right),$$

(29) $$u^{k+1}=\underset{u}{\arg\min}〈A^{*}p^{k+1},u〉+\lambda {\left\|u-f\right\|}_{1},$$

(30) $${\bar{u}}^{k+1}=u^{k+1}+\theta \left(u^{k+1}-u^{k}\right),\theta \in \left[0,1\right].$$

Similar to 35, 36, the maximization problem (28) has the closed‐form solution:

(28a) $$\begin{array}{l} \hat{p}=p^{k}+\tau_{1}A{\bar{u}}^{k},\\ p^{k+1}\left(i\right)=\frac{\hat{p}\left(i\right)}{\max\left(1+{\alpha \tau }_{1},|\hat{p}\left(i\right)|\right)}. \end{array}$$

where *τ*_1_ is the dual step size and *α* is defined in the Huber regularization in (14) and (23). For an intuitive understanding of (28a), we note that *J* ^*^ can be interpreted as the indicator function for the unit ball in the dual norm,

(31) $$J^{*}\left(p\right)=\left\{\begin{array}{cc} 0, & {\left\|p\right\|}_{*}\leq 1,\\ \infty , & \text{otherwise} \end{array}\right.$$

and then problem (28) is equivalent to solve the dual problem:

(32) $$p^{k+1}=\underset{p\in X}{\arg\max}〈p,A{\bar{u}}^{k}〉,$$

where *X* = {*p*, J^*^(*p*) ≤ 1}. Since the ascend direction of (32) is $A{\bar{u}}^{k}$, (28a) can be considered as updating *p* along the ascend direction and projecting *p* onto *X.*

We solve the primal problem (29) with the primal algorithm described in 35, where the L_1_ norm can be solved by the pointwise shrinkage operations:

(29a) $$\begin{array}{l} {\hat{u}}^{k+1}=u^{k}-\tau_{2}A^{*}p^{k+1},u^{k+1}\left(i\right)\\ =\left\{\begin{array}{ccc} \hat{u}\left(i\right)-\tau_{2}\lambda & \text{if} & \hat{u}\left(i\right)-f\left(i\right)>\tau_{2}\lambda ,\\ \hat{u}\left(i\right)+\tau_{2}\lambda & \text{if} & \hat{u}\left(i\right)-f\left(i\right)<-\tau_{2}\lambda ,\\ f\left(i\right) & \text{if} & |\hat{u}\left(i\right)-f\left(i\right)|\leq \tau_{2}\lambda . \end{array}\right. \end{array}$$

Here *τ*_2_ is the primal step size and the conjugate of *A* is:

(33) $$A^{*}p^{k+1}\left(i\right)=-\mathrm{div}\left(S{\left(i\right)}^{T}p^{k+1}\left(i\right)\right).$$

Problem (24) is convex and the efficiency of the proposed algorithm comes from the ability to find closed‐form solutions for each of the sub‐problems. We summarize the proposed algorithm, including the estimation of the diffusion tensor, in Algorithm 1. This algorithm is partially inspired by the work of Estellers et al. 36. Note that we use the forward differences to compute the discrete gradients and backward differences for the divergence to preserve their adjoint relationshipdiv =  − ∇^*^.

Algorithm 1 The efficient algorithm for the convex minimization problem (24).

**Initialization**. Set ${\bar{u}}^{0}=u^{0}=f,p^{0}=0,k=0.$ Choose the initial step size *τ*_1_, *τ*_2_ > 0 and *θ* ∈ [0, 1].

**While** (||*u* ^*k* + 1^ *− u* ^*k*^|| *> ε*).

1. Compute the structure tensor *J*, using (2).
2. Construct diffusion matrix *S*: in the flat areas, set *S* as the identity matrix; otherwise, compute *S* using (16).
3. Update *p*^*k*^, *u*^*k*^ and ${\bar{u}}^{k}$ iteratively, using (28a), (29a) and (30).
4. Set *k* = *k* + 1.

**Output:** *u* ^*k* + 1^, when ||*u* ^*k* + 1^ *− u* ^*k*^|| ≤ *ε* is satisfied.

## 4 EXPERIMENTAL RESULTS

We validate the proposed TRTV‐L_1_ model on a 2D simulated image, and around 200 2D and 3D THG images of normal human brain and tumor tissue. The field of view of the 2D and 3D THG images is 273 × 273 μm^2^ (1125 × 1125 pixels) and 273 × 273 × 50 μm^3^ (1125 × 1125 × 50 voxels), respectively. The intensities of these images are scaled to [0, 255]. We have previously developed a salient edge‐enhancing ADF model (the SEED model) to process the THG images of normal brain tissue 15, while the images of tumor tissue have not been published for the purpose of image analysis before. We compare our 2D results with the TV model 34, the edge‐enhancing ADF model (the EED model) 16, the BM3D model 62, the HS model 40, the STV model 38, the ATRTV model 36 and our previous SEED model 15. We only compare our 3D results with the TV model and the SEED model because not all source codes are readily available for other models in 3D. A comparison between EED and SEED has already been made in 15 for 3D.

### 4.1 Implementation

The proposed TRTV‐L_1_ model and ADFs are implemented in Visual Studio C++ 2010 on a PC with 8 3.40‐GHz Intel(R) Core(TM) 64 processors and 8 GB memory. Multiple cores have been used to implement the 3D algorithms, and a single core has been used for the 2D implementation. The TV model is implemented using the primal‐dual algorithm described in 34. The ADF models are implemented in the semi‐implicit scheme 17. The Matlab source codes for the BM3D model 62, the HS model 40, the STV model 38 and the ATRTV model 36 are available online from the authors' websites. The parameters are manually optimized for each model. The key parameters used for the proposed TRTV‐L_1_ model involve *λ* = 0.15, *τ*_1_ = 0.02, *τ*_2_ = 8.5 and *θ* = 1.0 for 2D and *λ* = 0.15, *τ*_1_ = 0.05, *τ*_2_ = 1.5and *θ* = 1.0 for 3D. The convergence accuracy *ε* is set to 10^−2^.

### 4.2 Denoising effect

**Figure 1:**
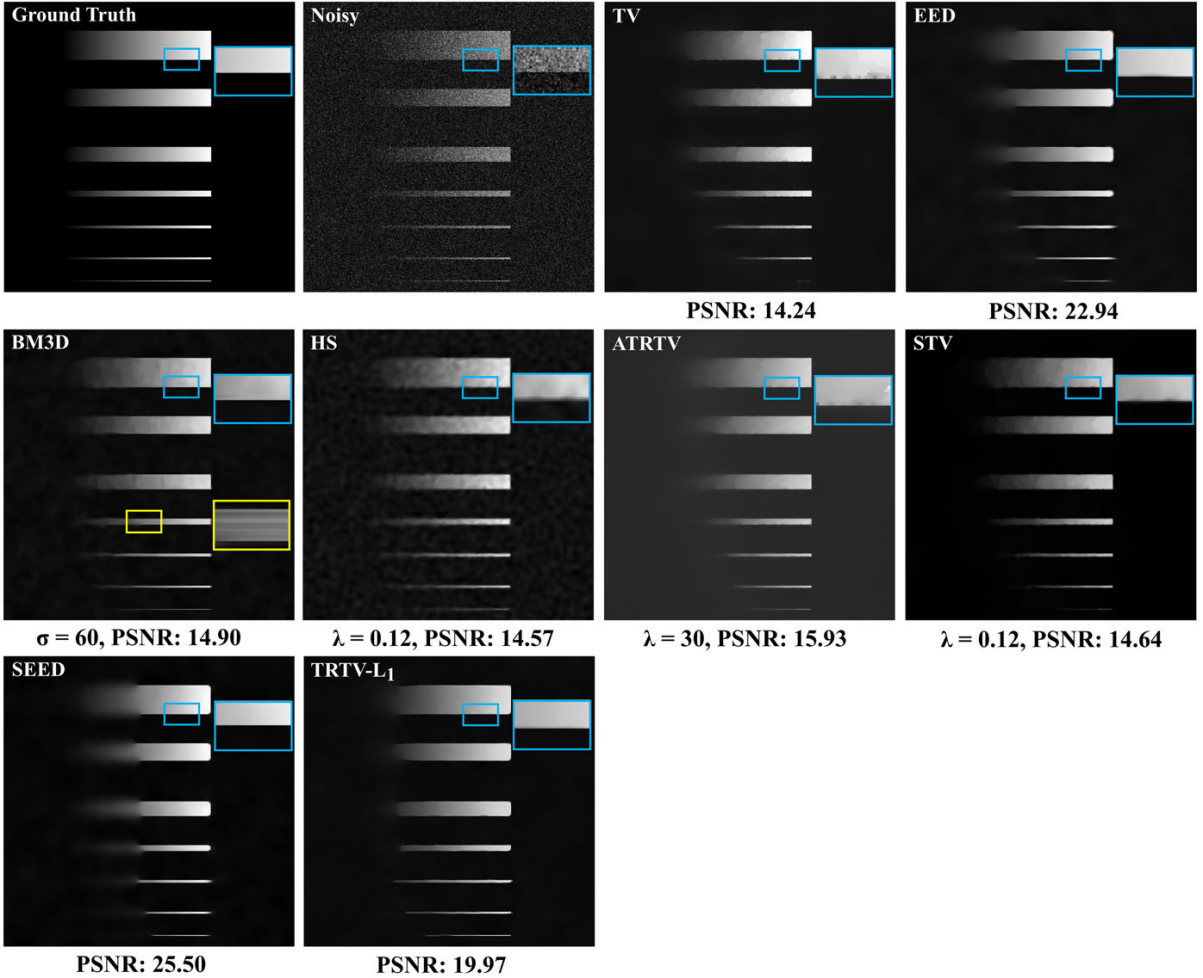
Comparison of denoising results on the simulated image

The performance of the proposed TRTV‐L_1_ model is first evaluated on a 2D simulated image (Figure 1). The simulated image consists of seven horizontal lines of the same width (255 pixels), but of different heights, 50, 30, 25, 10, 5, 3 and 1 pixels. The intensity of each line horizontally increases from 1 to 255, mimicking edges with varying gradients. Gaussian noise with SD of 60 is added to simulate strong noise. The TV model cannot remove the noise on the edges (blue square), creates *stair‐casing* effect, fails to restore the 1‐pixel line and restores the 3‐pixel line only partially. The ADF models, that is, the EED and SEED models, have the highest peak signal‐to‐noise ratio 36 and they provide the best denoising effect, but they also lose some weak edges of all the lines. The BM3D model has perfect performance on keeping fine details, for example, a large part of 1‐pixel line is kept, but it creates ripple‐like artifacts (yellow square) and its denoising effect is not comparable to the tensor methods. The HS model penalizes the second‐order derivatives and thus it is able to avoid the *stair‐casing* effect and capture blood‐vessel‐like structures, but it has limited denoising effect and creates dark‐dot‐like artifacts. The ATRTV model is able to get rid of most *stair‐casing* effect, but the noise on the edges (blue square) is not properly removed. This behavior remains for other parameter settings. A possible explanation could be that there is not enough diffusion strength along the edge direction, possibly caused by the design of diffusion tensor. Its ability of keeping fine details is also limited, for example, part of the 1‐pixel line is swiped out. STV suffers less *stair‐casing* effect than TV, but its performance on denoising and keeping fine details is also limited, because it does not consider the eigenvectors that are the key for restoring local structures. Our TRTV‐L_1_ model combines the benefits of the L_1_ norm and tensor regularization, and has a denoising performance that is comparable to the ADF models and higher than the other models. Moreover, TRTV‐L_1_ is also able to keep fine details as BM3D does. The weak edges of all the simulated lines are better restored by TRTV‐L_1_ than by other tensor methods and regularization methods.

**Figure 2:**
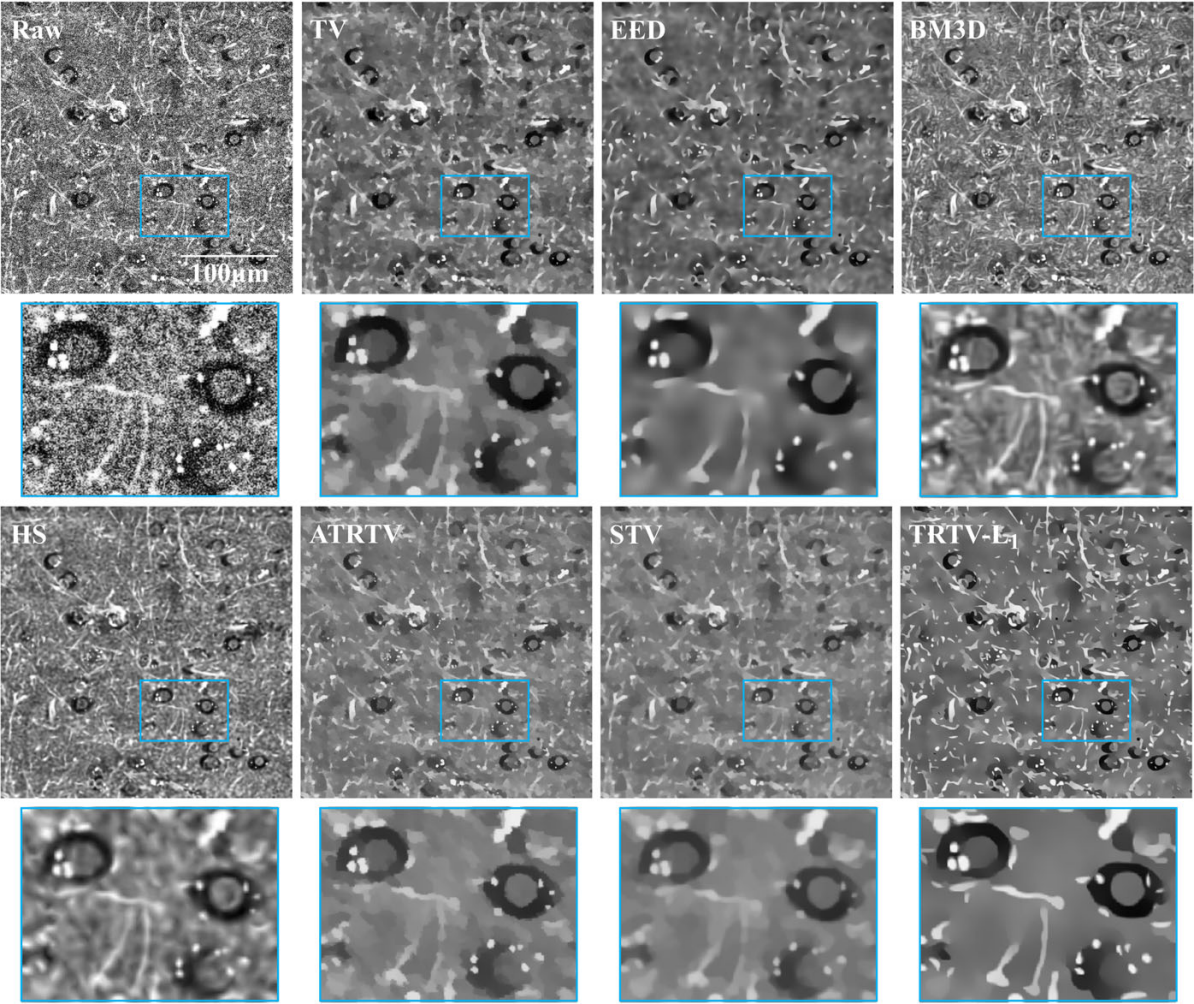
One 2D THG image of normal brain tissue from gray matter. Brain cells and neuropil appear as dark holes with dimly seen nuclei inside and bright fibers, respectively

We then compare the performance of the proposed TRTV‐L_1_ model with the aforementioned models using around 200 THG images of normal human brain and tumor tissue. One 2D typical example of THG images of normal brain tissue from gray matter is depicted in Figure 2. Brain cells (mainly including neurons and glial cells) and neuropil (consisting of axons and dendrites) are the basic features in a human brain, which appear as dark holes with dimly seen nuclei inside and bright fibers, respectively. Brain cells and neuropil are sparsely distributed in gray matter. The strong noise and rich morphologies contained in these THG images make the image denoising challenging. The TV model is able to remove the noise but it causes the *stair‐casing* effect. It cannot restore the thin fiber‐like neuropil because it does not consider the distribution of the local gradients (blue square). The ADF models (the SEED result is similar to the EED result and thus it is omitted) already give very satisfying results. The noise has been properly removed, but a substantial amount of weak edges have been smoothed to some extent, for example, the weak edges of some fibers and dark brain cells (blue square), because they are equivalent to the anisotropic TV with the L_2_ regularizer. The BM3D keeps the most fine details (the thin neuropil in the blue square), but its denoising effect is limited and again it creates ripple‐like artifacts. Note that the parameter *σ* involved in BM3D reflects the noise level of an image and the result of *σ* = 100 shown in Figure 2 indicates that the noise level of THG images is comparable to Gaussian noise with SD of 100. The HS model has limited performance on suppressing the strong noise in THG images, the result seems a bit blurred and dark‐dot‐like artifacts are created as appeared in the simulated image (Figure 1, HS). The result of ATRTV is similar to TV (but with less *stair‐casing* effect), and it is not able to restore the thin neuropil with weak edges. The STV model causes little *stair‐casing* effect, and does not keep fine details due to the lack of directional information. Compared with BM3D and HS, our TRTV‐L_1_ model is able to keep reasonable amount of fine details yet has a significantly superior denoising performance. Compared to other tensor and regularization methods, TRTV‐L_1_ can keep all salient edges and many more weak edges and fine details. TRTV‐L_1_ also provides the best image contrast and suffers almost no *stair‐casing* effect, because of the L_1_ norm and the robust anisotropic regularizer used. Results presented in Supporting Information (Figures S1‐S7) indicate that the parameter settings in Figure 2 are optimal for BM3D, HS, ATRTV and STV. The comparison of the segmentations resulted from the denoised images (Figures S5 and S6) not only confirms our qualitative evaluation of the denoising performance but also suggests that the denoising effort of TRTV‐L_1_ can really benefit the following segmentation step.

**Figure 3:**
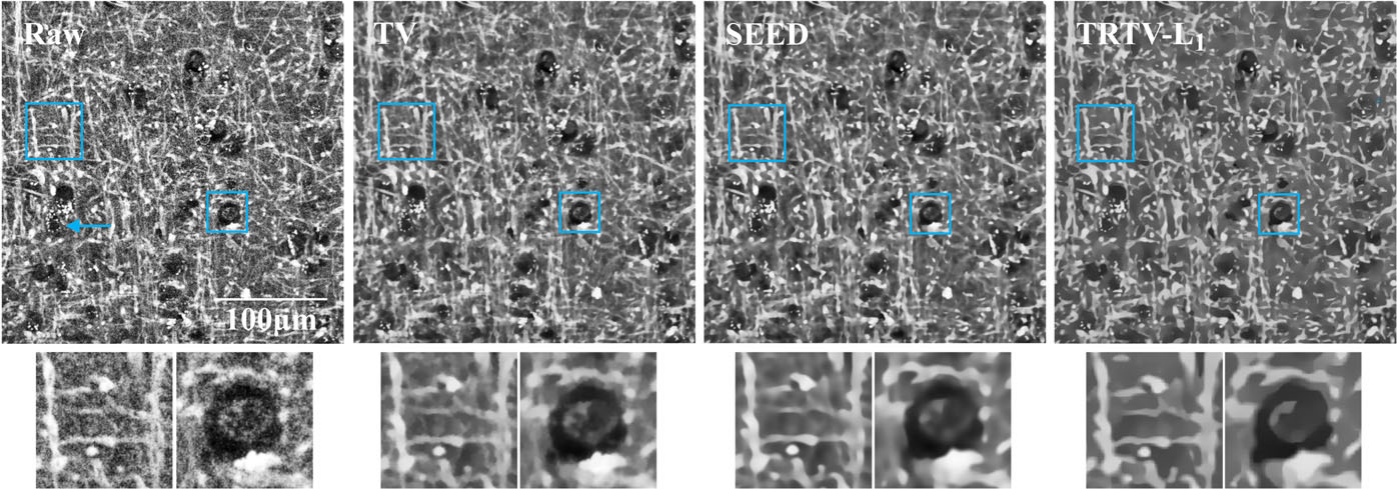
One 3D THG example of normal brain tissue from white matter, with the 33th slice shown. More neuropil is observed than in gray matter

3D THG images of normal brain tissue from white matter (Figure 3) are adequate testing materials to demonstrate the 3D performance of the proposed model, because of the presence of the complex morphologies, for example, nets of neuropil. The density of brain cells, for example, neurons with dimly seen nucleus or with lipofuscin granules inclusions (blue arrow), is low but the density of neuropil is higher than in gray matter. We see that the noise has been removed by all the models. Nevertheless, the TV model cannot enhance the fiber‐like structures (the left blue square) and suffers from the *stair‐casing* effect. The SEED model is able to enhance the fibers, but some weak edges have been in fact smoothed (the blue square). Only our TRTV‐L_1_ model succeeds to reconstruct almost all the sharp and weak edges.

**Figure 4:**
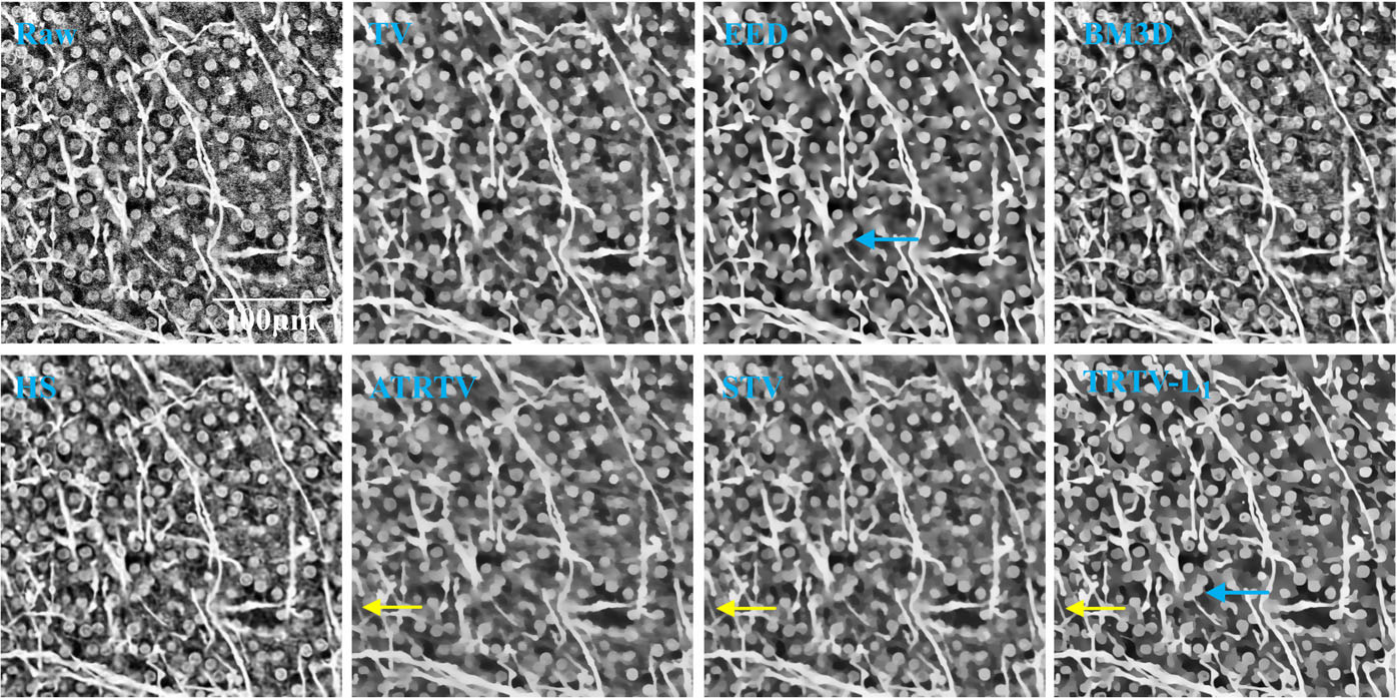
One 2D THG example of low‐grade tumor tissue from an oligodendroglioma patient. High cell density and thick neuropil indicate the presence of a tumor

One 2D example of THG images of the low‐grade tumor tissue obtained from an oligodendroglioma patient is shown in Figure 4. Compared to the THG images of normal brain tissue, more brain cells (including cell nuclei and the surrounding cytoplasm) are present that indicates the presence of a tumor. Again, the TV model suffers from the *stair‐casing* effect. The ADF models fail to restore the weak edges (blue arrow). BM3D and HS have weaker denoising effect than other methods. BM3D creates ripple‐like artifacts and HS blurs the image. In contrast to the conventional approach for tensor estimation, ATRTV attempts to capture the directionality and scale of local structures via another convex approximation, but our results on THG images do not suggest superior merits of this aspect of ATRTV over the conventional approach in restoring local structures. The result of STV is similar to that of ATRTV due to the lack of directional information. Compared to other models, TRTV‐L_1_ either has better denoising performance and/or restores more fine details and weak edges (blue and yellow arrows).

**Figure 5:**
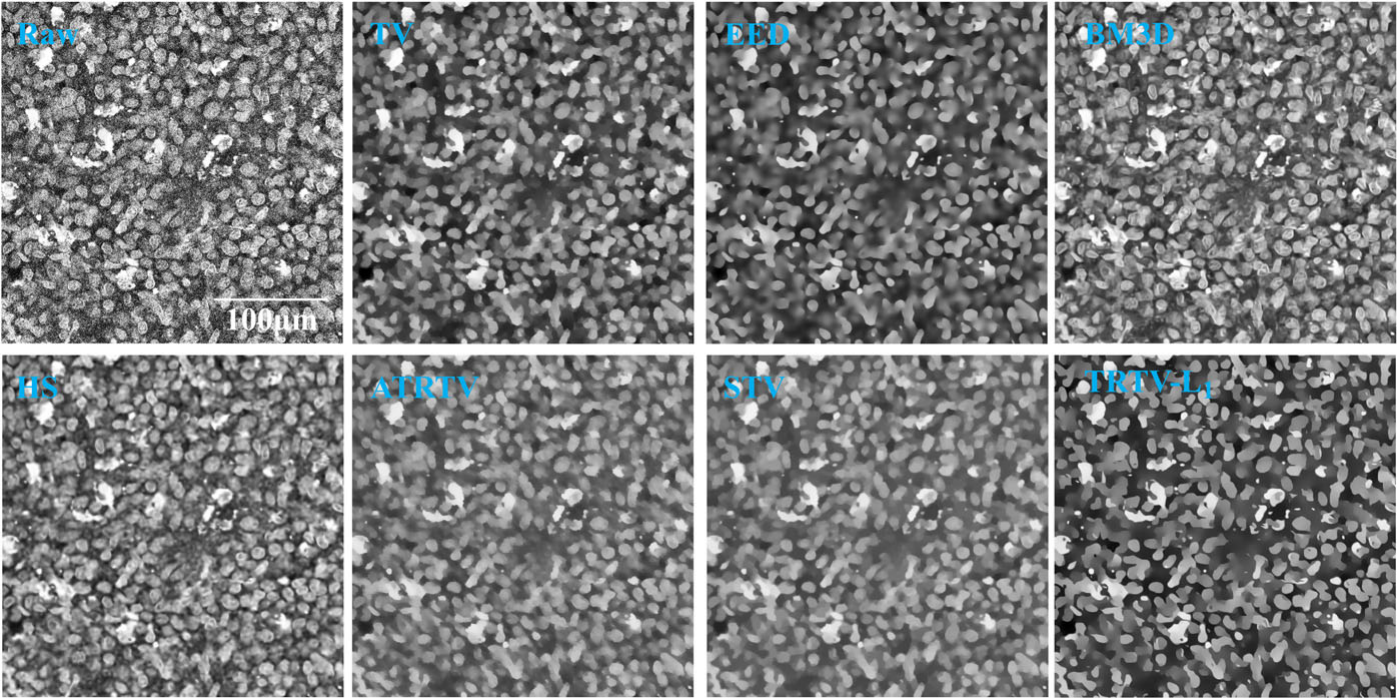
One 2D THG example of high‐grade tumor tissue from a glioblastoma patient. The whole area is occupied by tumor cells

One 2D example of THG images of the high‐grade tumor tissue obtained from a glioblastoma patient is shown in Figure 5. All the fiber‐like neuropil are now completely absent and the whole area is filled with cell nuclei. The density of cell nuclei here is even higher than that of the low‐grade tumor tissue, indicating that those cells likely represent tumor cells. The TV model is able to reconstruct both the salient and weak edges but it again causes the *stair‐casing* effect around the edges. The ADF models provide quite similar results without any *stair‐casing* effect, but the weak edges have been blurred. BM3D and HS have limited denoising effect. ATRTV and STV suffer less *stair‐casing* effect than TV, but the contrast seems degenerated. The proposed TRTV‐L_1_ model has reconstructed the salient and weak edges, which will greatly facilitate applications like automatic cell counting.

### 4.3 Computational performance

**Figure 6:**
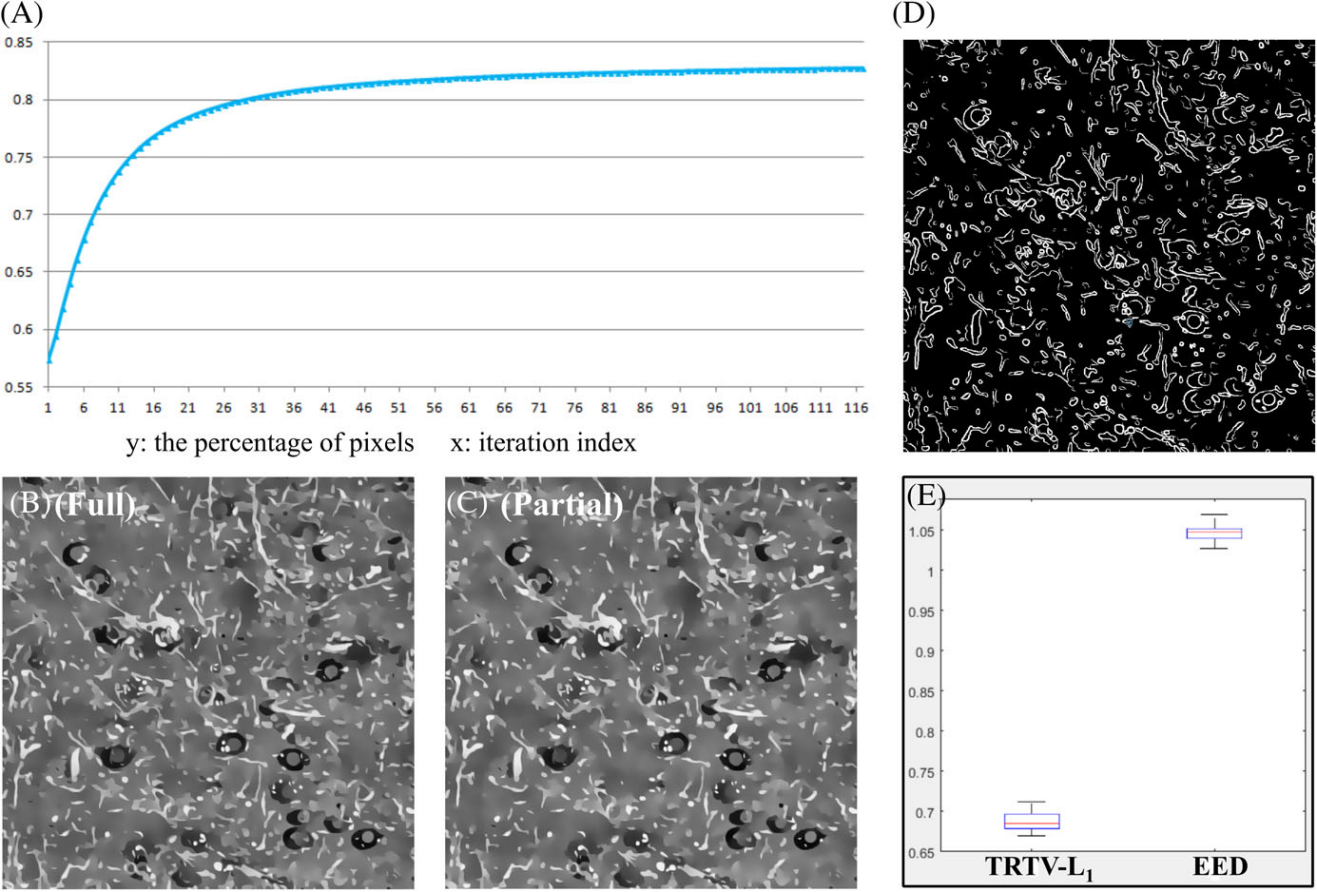
Computational performance of the proposed TRTV‐L_1_ model. (A) The percentage of pixels (*y*‐axis) that are considered as points in the flat regions, in each iteration (*x*‐axis) during the tensor decomposition of a 2D THG image. (B, C) Comparison of the TRTV‐L_1_ results with (B) and without (C) full tensor decomposition everywhere. (D) The visual map of non‐flat regions that results from the last iterative step. (E) The time per iteration (*y*‐axis) needed for TRTV‐L_1_ and EED, tested on the 30 2D THG images

We first evaluate the computational cost of TRTV‐L_1_ that has been saved by restricting the tensor decomposition to the non‐flat areas. Roughly, the flat regions estimated in each iteration increase from 50% up to 90% of the whole image domain, and on average, 80% pixels are considered as flat regions, indicating that tensor decomposition is needed only for 20% of the pixels (Figure 6A). The reconstruction with and without full estimation of the structure tensor everywhere has been compared using THG images, from the aspects of timing and restoration quality. No significant difference in the number of iterations needed for convergence is observed between the full and partial estimation of structure tensor. For 2D THG images the partial estimation approach saves ~10% of computation time, either in terms of convergence time or time per iteration. No significant degradation has been found in the restoration quality (Figure 6B,C and Figure S7) when *h* varies from 0.0 to 0.9, and thus we use *h* = 0.9 to obtain maximal gain in speed. We also find that the absolution difference per pixel between the two reconstructions is 3.8, indicating the small difference between the 2 solutions. As a reference, the absolution difference per pixel between the reconstruction using partial estimation of structure tensor and the input noisy image is 54.4. For 3D THG images, ~40% of computation time is saved by the partial estimation approach. A visual map of non‐flat regions that results from the last iterative step is shown in Figure 6D. This map actually consists of all the sharp edges of the image, which conversely suggests that the weak edges are restored from the regularization and L_1_ fidelity rather than from the diffusion. Similar tests on the EED model indicate that the same computational gains can be achieved for the ADF models, using the partial tensor decomposition.

To demonstrate the computational efficiency of the proposed TRTV‐L_1_ model, we compare the average computational time needed by TRTV‐L_1_ to the ADF models on 30 2D and 5 3D THG images. The semi‐implicit scheme used to implement ADFs allows larger time steps than the explicit scheme. We found that ADFs converge much slower and consume more time per iteration than TRTV‐L_1_. For example, TRTV‐L_1_ on average needs only ~1/3 number of iterations of EED to converge to the same accuracy 10^−2^ and TRTV‐L_1_ consumes ~2/3 time of EED per iteration (Figure 6E), which results in a ~75% higher speed than EED. In practice, a fixed number of iteration is also a strategy to stop the iterations, and we find that both ADFs and TRTV‐L_1_ have already produced quite satisfying results after 50 iterations. In this condition, TRTV‐L_1_ is on average ~30% more time efficient than ADFs on 2D THG images, and ~60% more time efficient on 3D THG images. Compared to other tensor regularization models, our TRTV‐L_1_ model is roughly as efficient as the STV model, faster than the ATRTV model that uses another convex optimization to estimate structure tensor.

## 5 DISCUSSION AND CONCLUSIONS

In this work, we have developed a robust and efficient TRTV‐L_1_ model to restore images corrupted by noise. THG images of structurally normal human brain and tumor tissue have been tested. The proposed model showed impressively better results on the reconstruction of weak edges and fine details and it was more efficient than existing ADF and TRTV models. Comparisons to other state‐of‐art denoising techniques that are able to keep fine details, indicate that TRTV‐L_1_ can restore a reasonable amount of fine details but it has significantly better denoising performance without creating artifacts. The artifacts created by other models may result in false positives in subsequent segmentation steps. Therefore, the proposed TRTV‐L_1_ model will greatly facilitate the following segmentation and cell counting of THG images of brain tumor, from which we conclude that the robust and efficient TRTV model will strengthen the clinical potential of the THG microscopy on brain tumor surgery. Moreover, based on the tests on the simulated image and the THG images with complex morphologies, we believe that the proposed method can be generalized to other application‐driven projects. The efficient estimation of the diffusion tensor we proposed here can be used to accelerate most of the existing tensor diffusion and regularization models, by performing tensor decomposition only in the non‐flat regions. Compared to existing TRTV models, for example, the ATRTV model, the approach we combined the diffusion tensor and TV can be easily used to derive other application‐driven TRTV models from existing ADF models. The L_1_ norm in the data fidelity term makes the proposed TRTV‐L_1_ model contrast invariant, robust to noise and sensitive to fine details. The primal‐dual algorithm used to optimize the proposed model is easy‐to‐code in comparison with the existing ADF models because no sparse matrix inversions are involved. Although there are many other important types of image denoising methods as aforementioned, in this study we emphasize the benefits of tensor‐based techniques that they are able to capture local structures. Compared to other alternative approaches, for example, the machine learning methods, a training step is usually included which needs a training set of clear images with high SNR, but such images are difficult to acquire for THG brain images.

## AUTHOR BIOGRAPHIES

Please see Supporting Information online.

## Supporting information


**Author Biographies**

**Figure S1** Results of BM3D for *σ* = 50, 100 and 150. The denoising effect of BM3D increases with *σ.* The denoising effect starts to occur when *σ* = 50, and achieves its optimal effect for *σ* = 100. Larger *σ* does not contribute to further improvement. These images show that BM3D creates ripple‐like artifacts and has limited denoising performance.
**Figure S2** Results of Hessian Schatten‐Norm regularization (HS) for *λ* = 0.1, 0.3 and 0.5. The denoising effect starts to occur when *λ* = 0.1, and achieves its optimal performance for *λ* = 0.3. The result becomes too blurred when *λ* = 0.5. HS creates dark‐dot artifacts, has limited denoising performance and blurs the image.
**Figure S3** Results of ATRTV for *λ* = 18, 10, 5 and *μ* = 8.6, 5.0 and 3.0. ATRTV has better denoising effect when *λ* and *μ* are small. The denoising effect starts to occur when *λ* = 18, *μ* = 8.6, and achieves its optimal performance for *λ* = 10, *μ* = 5.0. The result becomes blurred when *λ* and *μ* get smaller. The result of ATRTV is similar to that of TV, with less *stair‐casing* effect created, but it is not able to restore fine details and weak edges corrupted by strong noise.
**Figure S4** Results of STV for *λ* = 0.24, 0.32 and 0.4. The denoising effect starts to occur when *λ* = 0.24, and achieves its optimal performance for *λ* = 0.4. The result of STV is similar to that of TV, with less *stair‐casing* effect created, but it is not able to restore fine details and weak edges corrupted by strong noise.
**Figure S5** Segmentations of the dark holes (brain cells) within the raw image and the denoised images in Figure 2, using manually optimized thresholds to detect most parts of the dark holes with least background included. The segmentation of the raw image indicates the strong noise present in the THG image. The segmentations of TV, EED, ATRTV, STV and TRTV‐L_1_ are similar but the small objects resident in segmentations of BM3D and HS illustrate their poor denoising performance.
**Figure S6** Segmentations of the bright objects (neuropil) within the raw image and the denoised images in Figure 2, using manually optimized thresholds to detect most parts of the bright objects with least background included (eg, the fiber indicated by yellow arrow). The segmentation of the raw image indicates the strong noise present. The segmentation of TRTV‐L_1_ is comparable to those of BM3D and HS, where more fibers have been resolved than other models. Sometimes fibers (blue arrows) are even better segmented from the image denoised by TRTV‐L_1_, which suggests that BM3D and HS could visually keep more details than TRTV‐L_1_ but it is not necessarily beneficial for the segmentation followed.
**Figure S7** Results of the proposed TRTV‐L_1_ model for *h* = 0.0 (full estimation), 0.2, 0.5, 0.8 and 0.9 (partial estimation). Almost no degradation has been found in the restoration quality when *h* varies from 0.0 to 0.9, and thus we use *h* = 0.9.
