# Supplementary material for: Tensor regularized total variation for denoising of third harmonic generation images of brain tumors
Source: J Biophotonics. 2018 Aug 16;12(1):e201800129. doi: 10.1002/jbio.201800129 (PMC7065612; doi:10.1002/jbio.201800129)
Supplement: Supplementary file 1 — Author Biographies [file JBIO-12-e201800129-s002.docx]

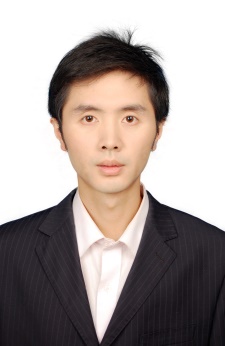


Zhiqing Zhang graduated in Mathematics at the Beijing Normal University in 2011. He obtained his PhD in 2017 at the Physics department of VU University Amsterdam, where he is now doing his postdoctoral research. His research interests include label-free microscopic imaging, microscopic image processing and machine learning, mathematical modelling, and convex optimization.


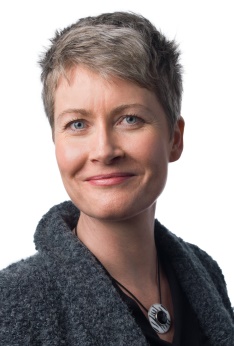
Marie Louise Groot obtained her PhD in 1997 in Biophysics at VU University Amsterdam, where she is appointed as a full professor. She performed postdoctoral research at the University of Chicago and at the Ecole Normal Superieure de Techniques Avancees. Upon return to VU University she set up a research line to study the structure-function relationship in proteins using femtosecond visible/midinfrared pump-probe spectroscopy. In 2010 she initiated a new research line to study live cell process in deep tissue. The developed second and third harmonic generation (SHG/THG) microscopy enables research with fundamental and pre-clinical aspects. She has co-authored 71 papers.


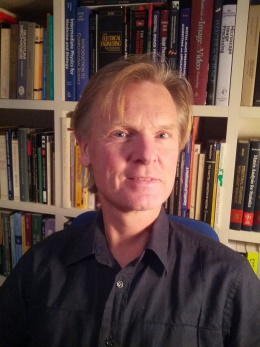
Jan C de Munck studied physics and obtained his PhD-degree in 1989 from the University of Amsterdam. De Munck is currently appointed as associate professor at VUmc. He is specialized in image and signal processing techniques applied to the clinical domain of neurological diseases. He has co-authored more than 100 scientific papers and he published the software developed during several research projects on the web at [http://demunck.info/software/](https://webmail.login.vu.nl/OWA/UrlBlockedError.aspx).
