# Supplementary material for: Tensor regularized total variation for denoising of third harmonic generation images of brain tumors
Source: J Biophotonics. 2018 Aug 16;12(1):e201800129. doi: 10.1002/jbio.201800129 (PMC7065612; doi:10.1002/jbio.201800129)
Supplement: Supplementary file 2 — Figure S1 Results of BM3D for σ = 50, 100 and 150. The denoising effect of BM3D increases with σ. The denoising effect starts to occur when σ = 50, and achieves its optimal effect for σ = 100. Larger σ does not contribute to further improvement. These images show that BM3D creates ripple‐like artifacts and has limited denoising performance. Figure S2 Results of Hessian Schatten‐Norm regularization (HS) for λ = 0.1, 0.3 and 0.5. The denoising effect starts to occur when λ = 0.1, and achieves its optimal performance for λ = 0.3. The result becomes too blurred when λ = 0.5. HS creates dark‐dot artifacts, has limited denoising performance and blurs the image. Figure S3 Results of ATRTV for λ = 18, 10, 5 and μ = 8.6, 5.0 and 3.0. ATRTV has better denoising effect when λ and μ are small. The denoising effect starts to occur when λ = 18, μ = 8.6, and achieves its optimal performance for λ = 10, μ = 5.0. The result becomes blurred when λ and μ get smaller. The result of ATRTV is similar to that of TV, with less stair‐casing effect created, but it is not able to restore fine details and weak edges corrupted by strong noise. Figure S4 Results of STV for λ = 0.24, 0.32 and 0.4. The denoising effect starts to occur when λ = 0.24, and achieves its optimal performance for λ = 0.4. The result of STV is similar to that of TV, with less stair‐casing effect created, but it is not able to restore fine details and weak edges corrupted by strong noise. Figure S5 Segmentations of the dark holes (brain cells) within the raw image and the denoised images in Figure 2, using manually optimized thresholds to detect most parts of the dark holes with least background included. The segmentation of the raw image indicates the strong noise present in the THG image. The segmentations of TV, EED, ATRTV, STV and TRTV‐L1 are similar but the small objects resident in segmentations of BM3D and HS illustrate their poor denoising performance. Figure S6 Segmentations of the bright objects (neuropil) within [file JBIO-12-e201800129-s001.docx]

Tensor regularized total variation for denoising of third harmonic generation images of brain tumors--supplementary material


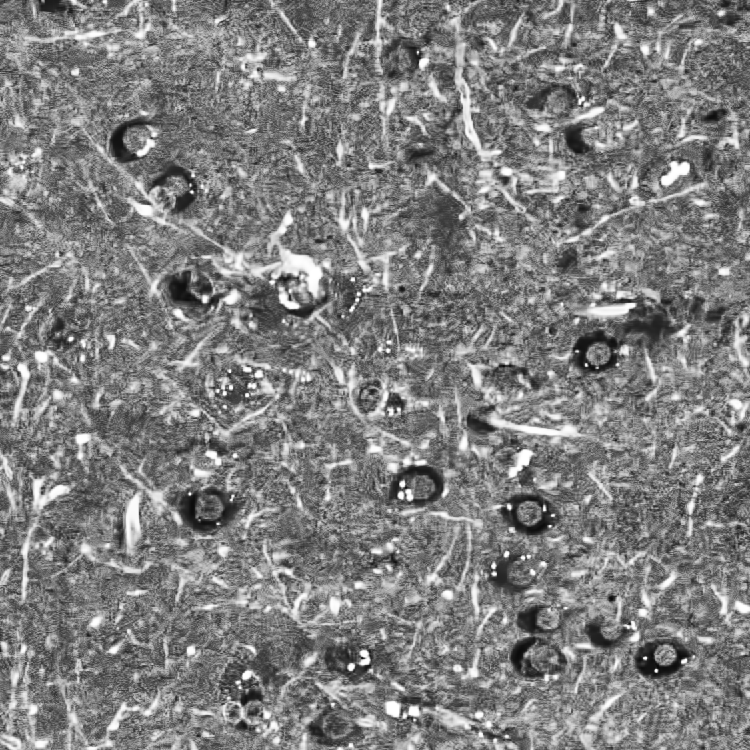

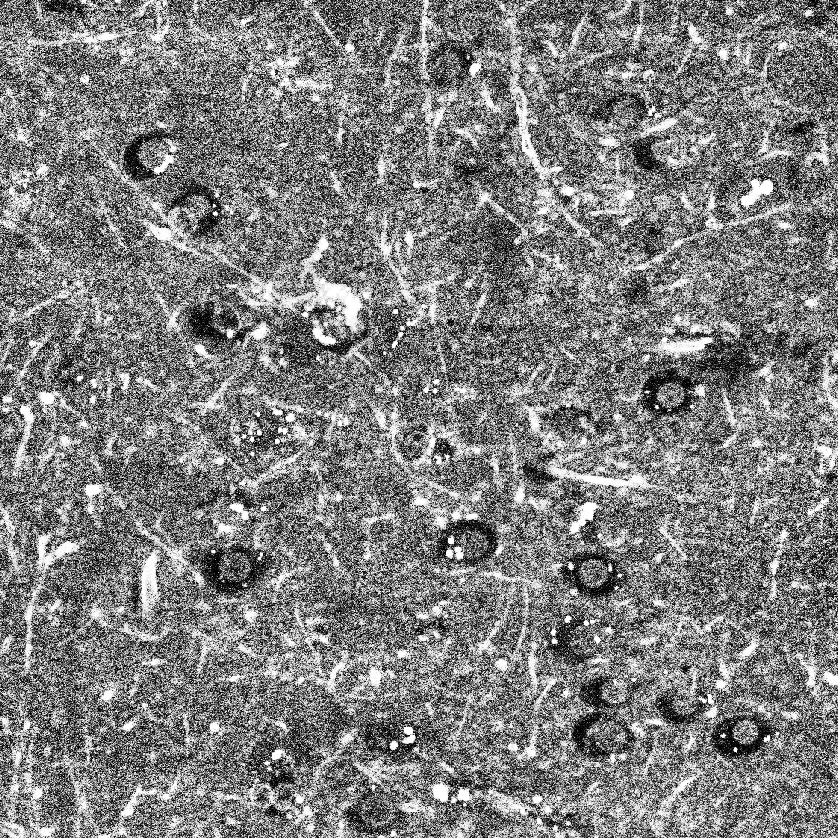


**Raw**

***σ* = 50**


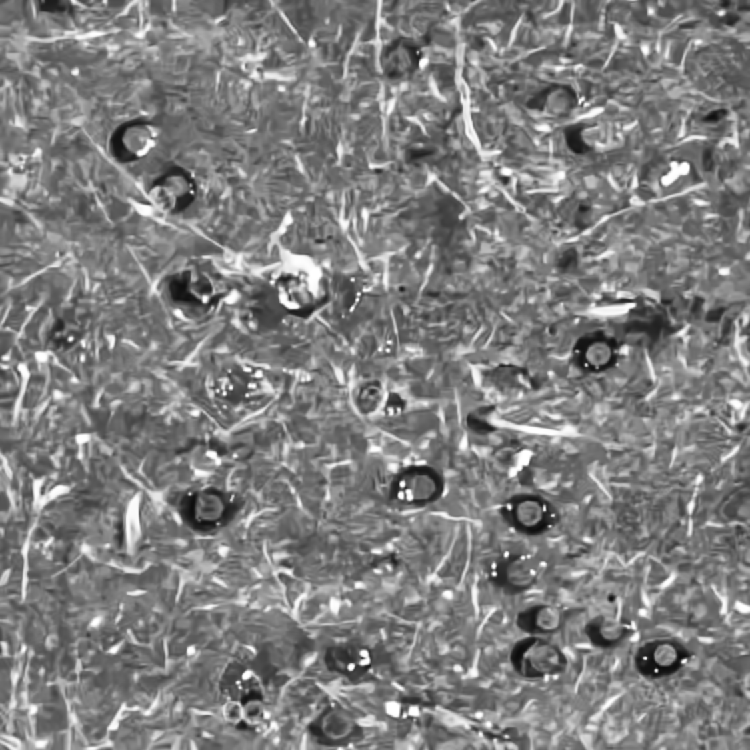


***σ* = 100**


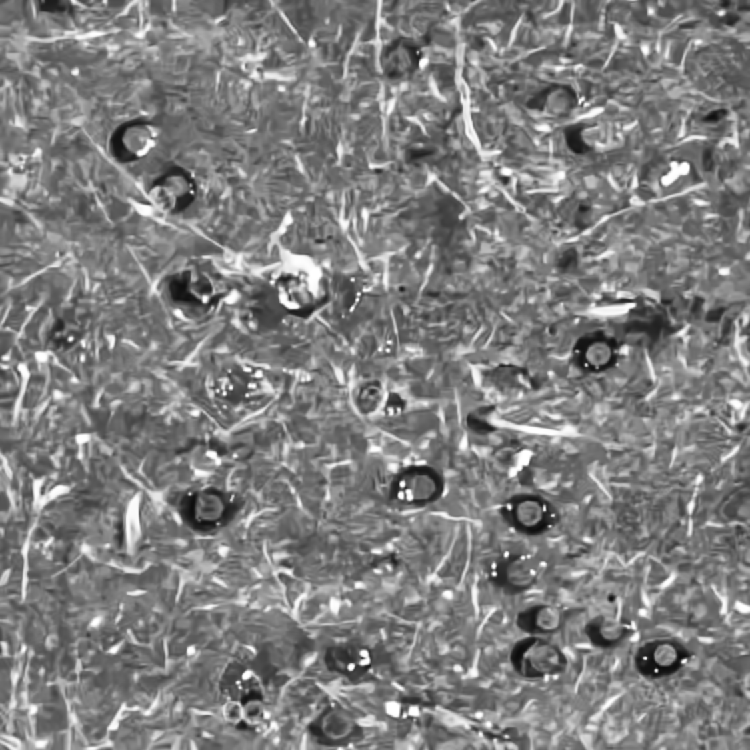


***σ* = 150**

S1. Results of BM3D for σ = 50, 100 and 150. The denoising effect of BM3D increases with σ. The denoising effect starts to occur when σ = 50, and achieves its optimal effect for σ = 100. Larger σ does not contribute to further improvement. These images show that BM3D creates ripple-like artifacts and has limited denoising performance.


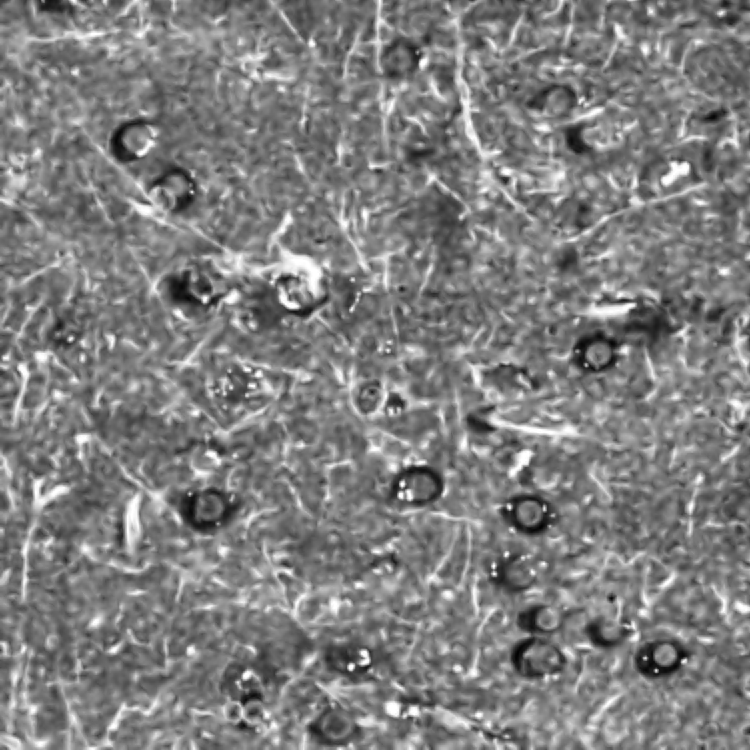

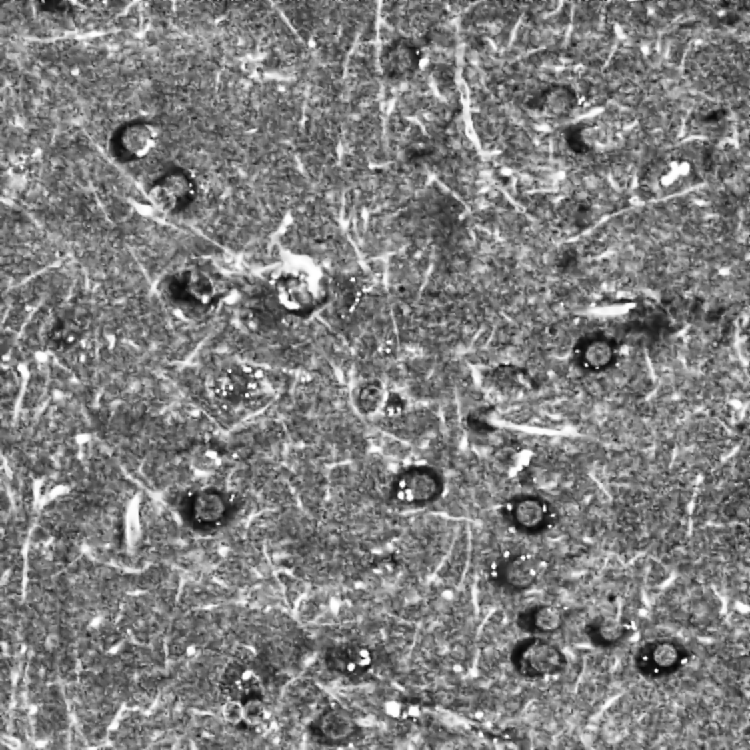

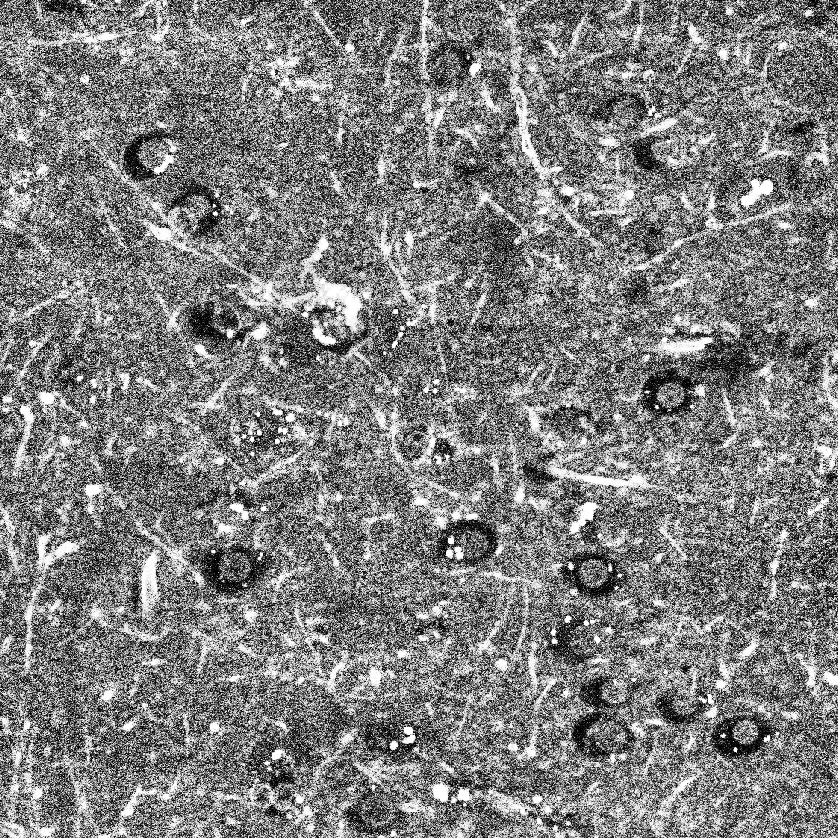


**Raw**

**λ = 0.1**

**λ = 0.3**


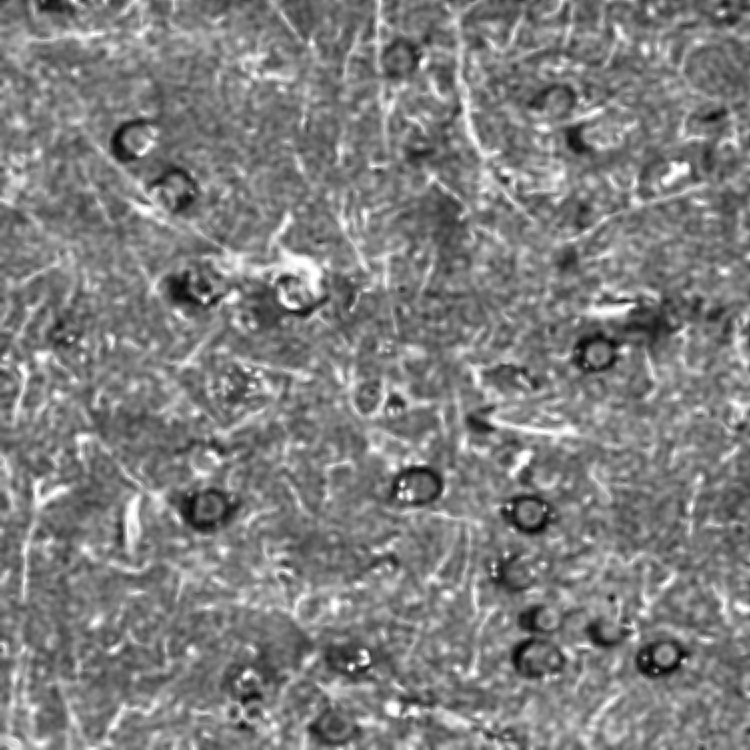


**λ = 0.5**

S2. Results of Hessian Schatten-Norm regularization (HS) for λ = 0.1, 0.3 and 0.5. The denoising effect starts to occur when λ = 0.1, and achieves its optimal performance for λ = 0.3. The result becomes too blurred when λ = 0.5. HS creates dark-dot artifacts, has limited denoising performance and blurs the image.


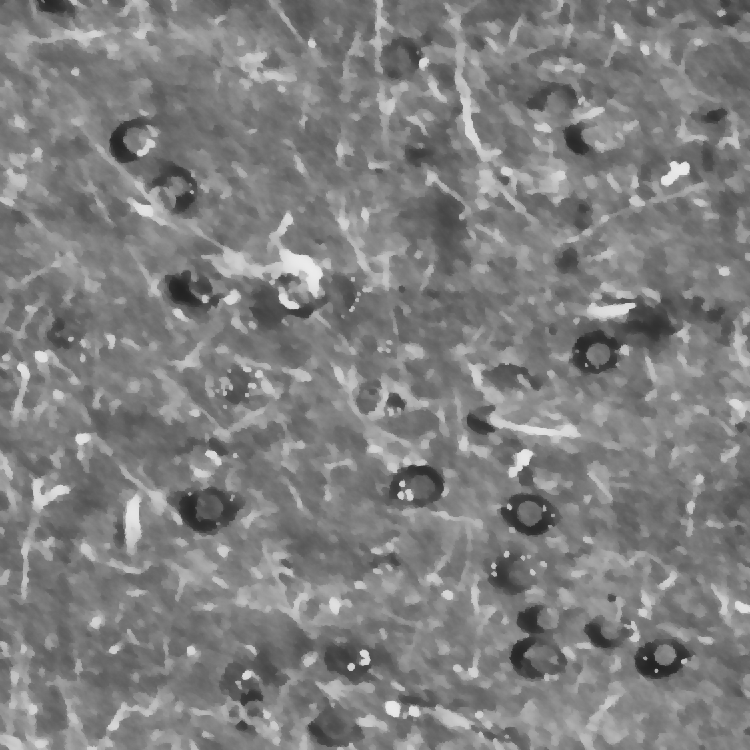

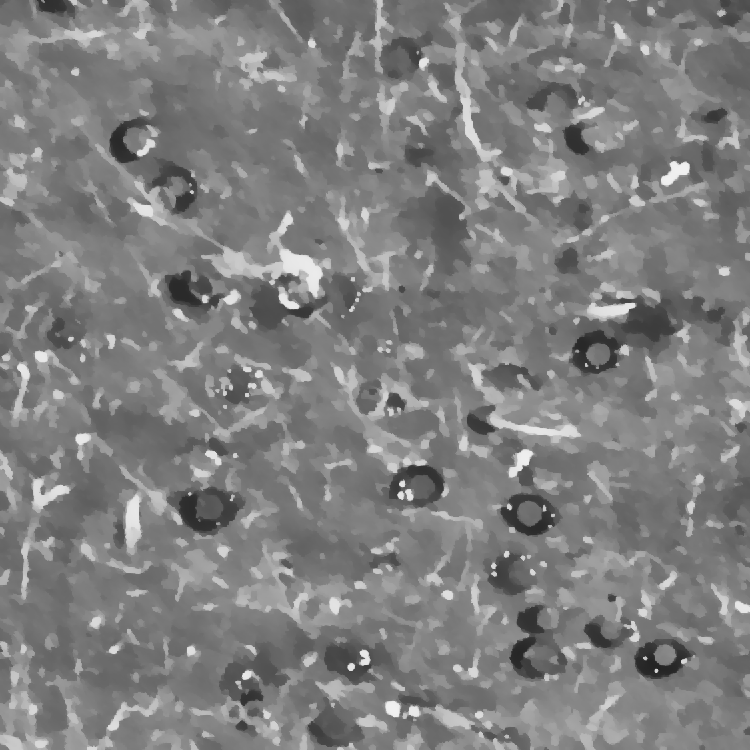

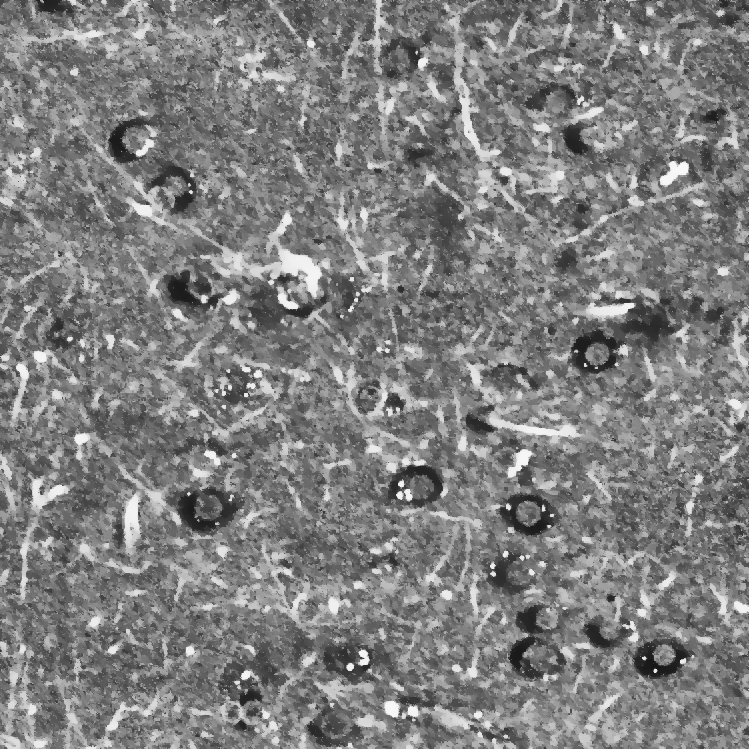

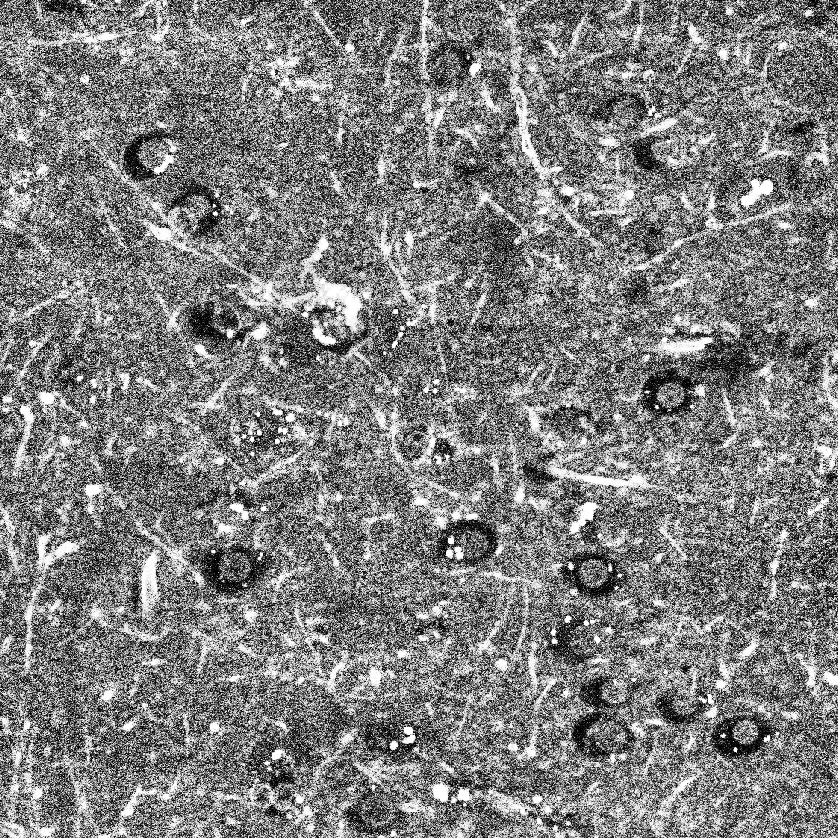


**Raw**

**λ = 18, µ=8.6**

**λ = 10, µ=5.0**

**λ = 5, µ=3.0**

S3. Results of ATRTV for λ = 18, 10, 5 and µ=8.6, 5.0, 3.0. ATRTV has better denoising effect when λ and µ are small. The denoising effect starts to occur when λ = 18, µ=8.6, and achieves its optimal performance for λ = 10, µ=5.0. The result becomes blurred when λ and µ get smaller. The result of ATRTV is similar to that of TV, with less *stair-casing* effect created, but it is not able to restore fine details and weak edges corrupted by strong noise.


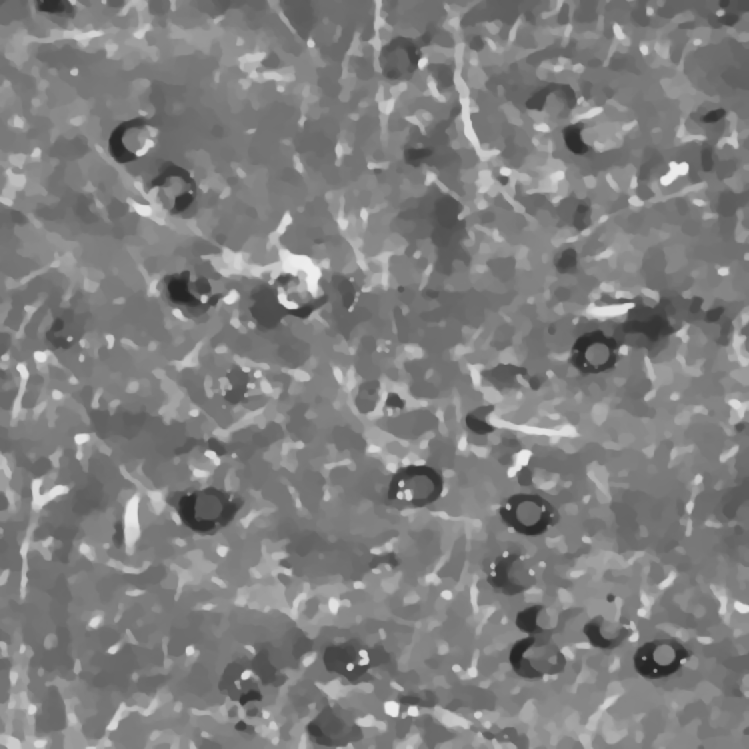

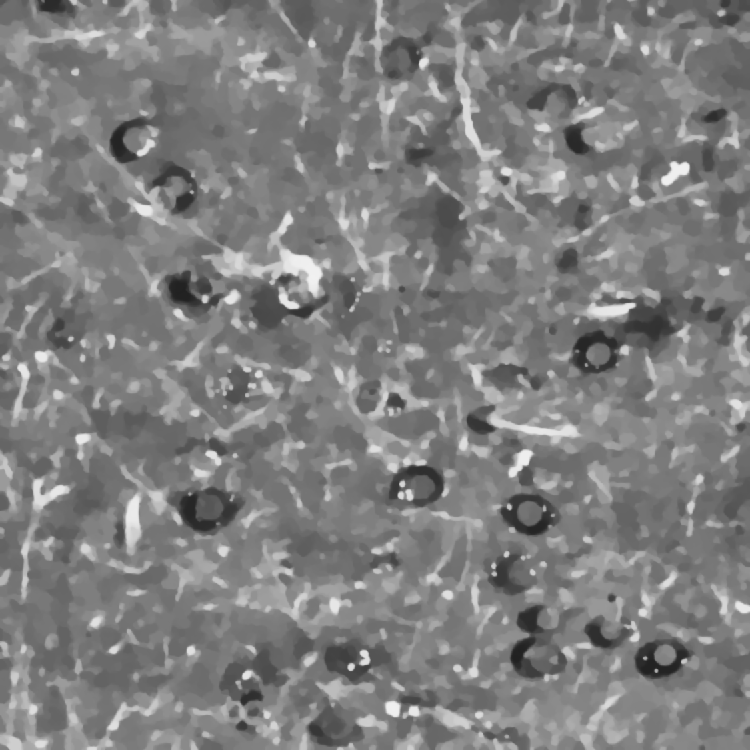

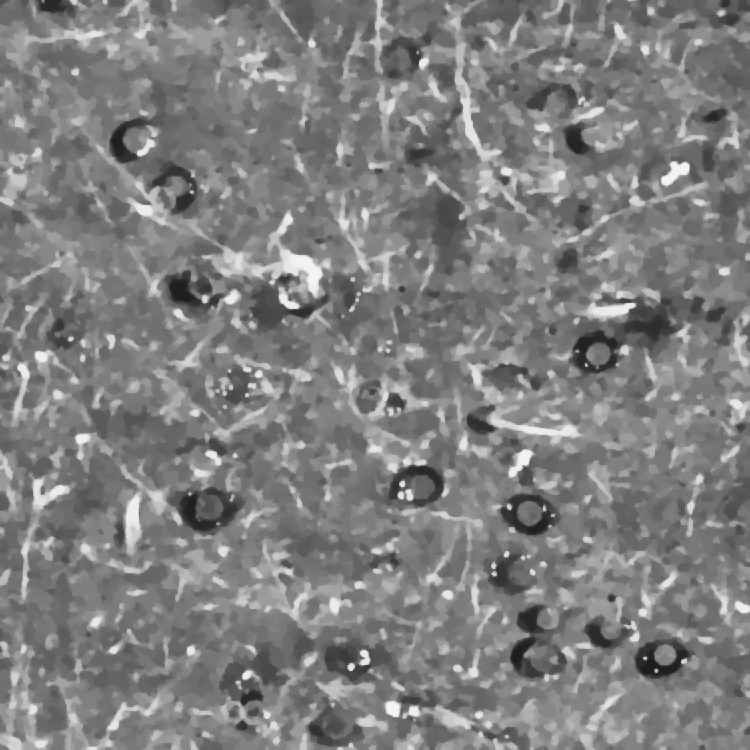

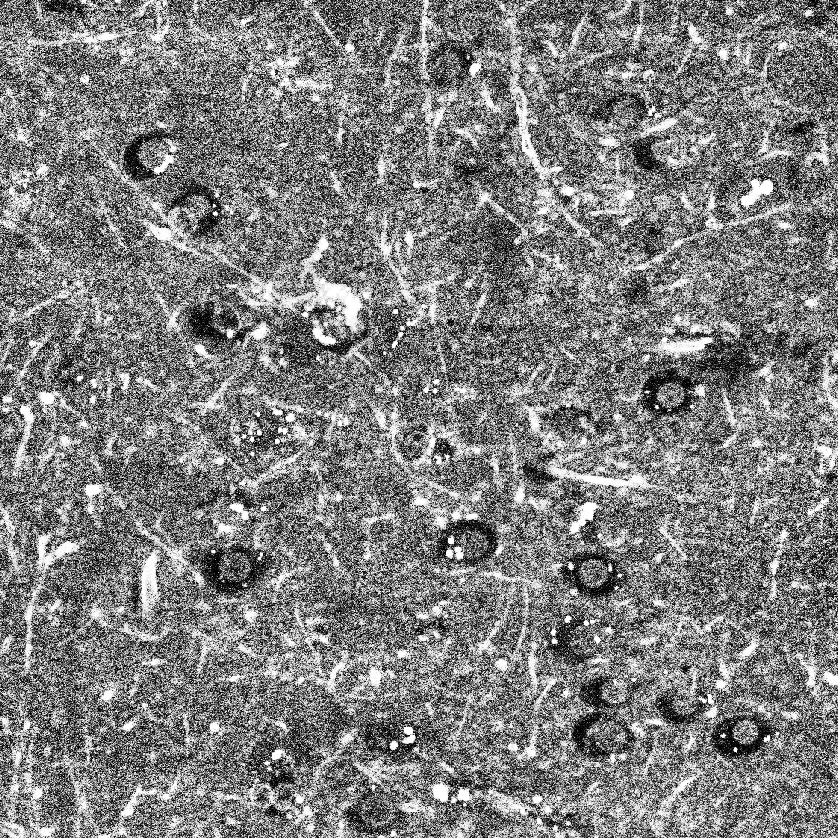


**Raw**

**λ = 0.24**

**λ = 0.32**

**λ = 0.4**

S4. Results of STV for λ = 0.24, 0.32 and 0.4. The denoising effect starts to occur when λ = 0.24, and achieves its optimal performance for λ = 0.4. The result of STV is similar to that of TV, with less *stair-casing* effect created, but it is not able to restore fine details and weak edges corrupted by strong noise.


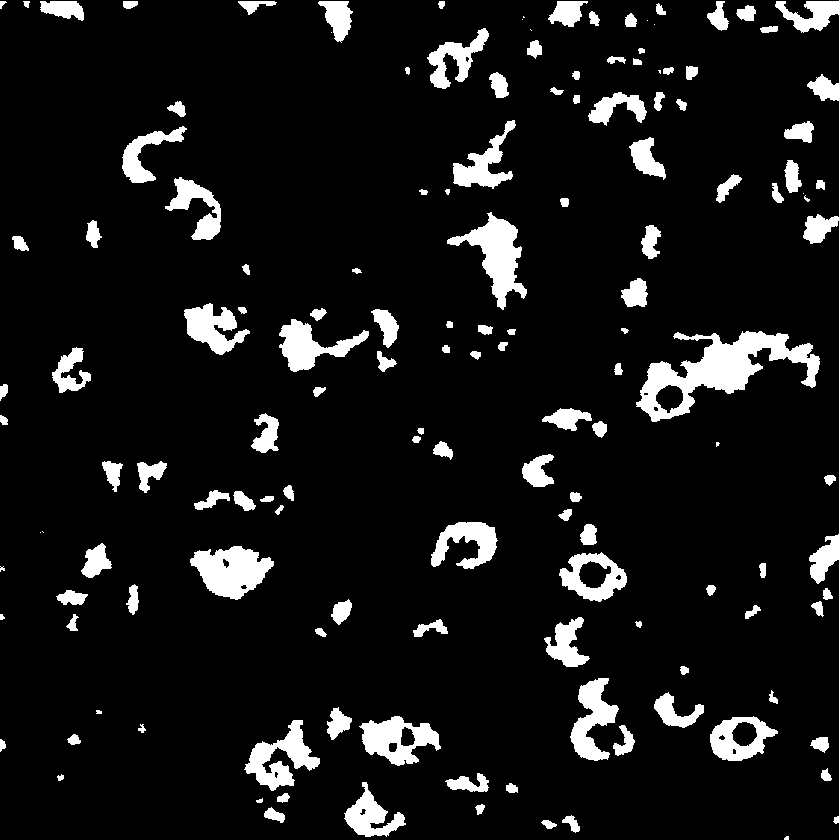

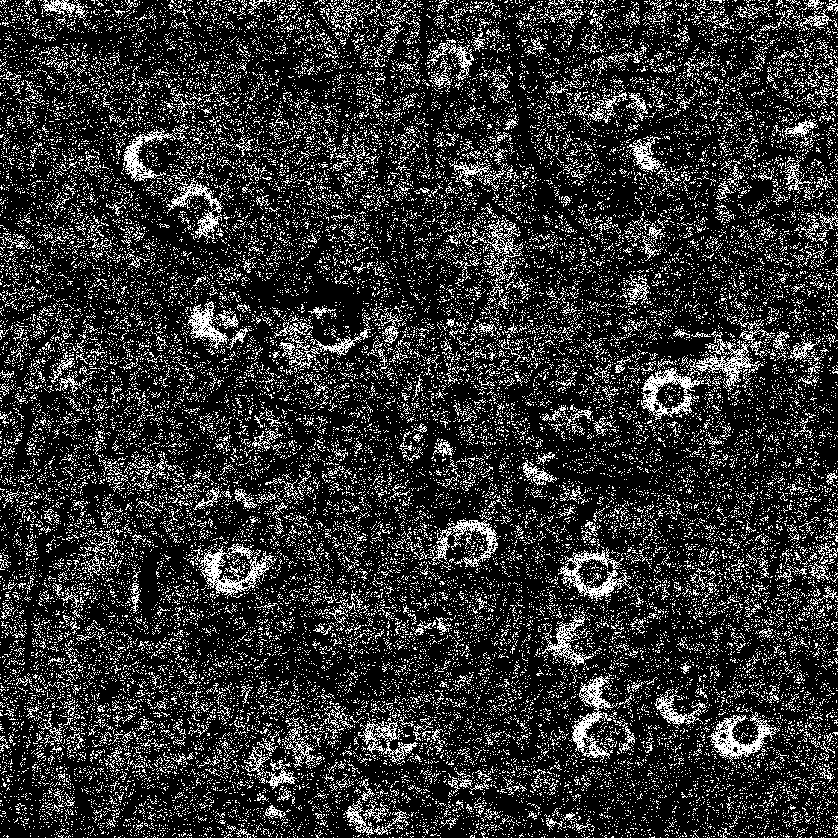


**Raw**

**TV**


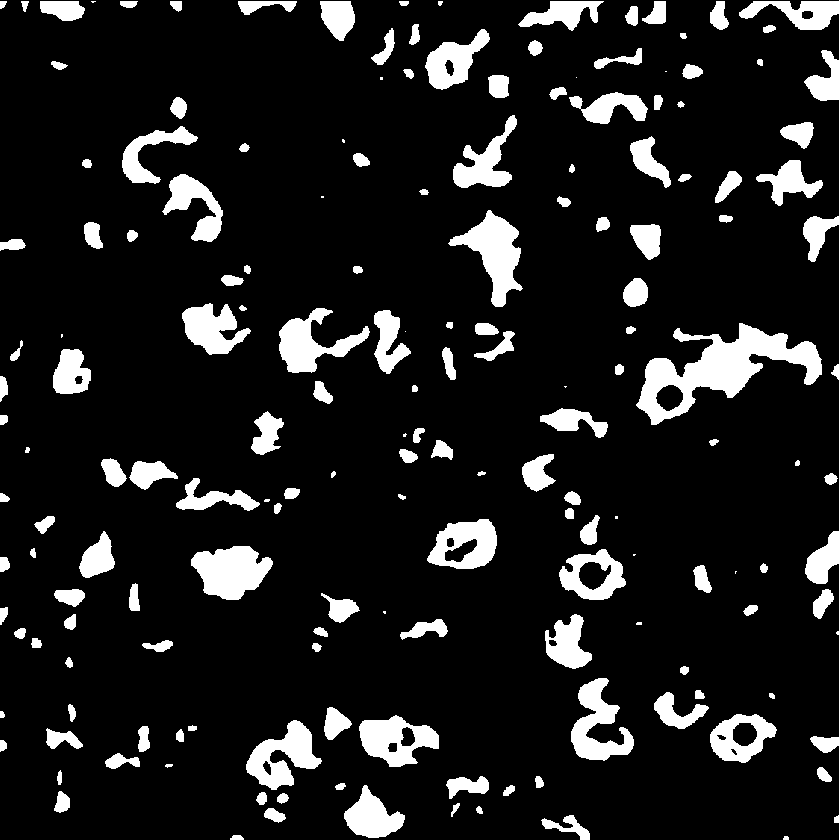


**EED**


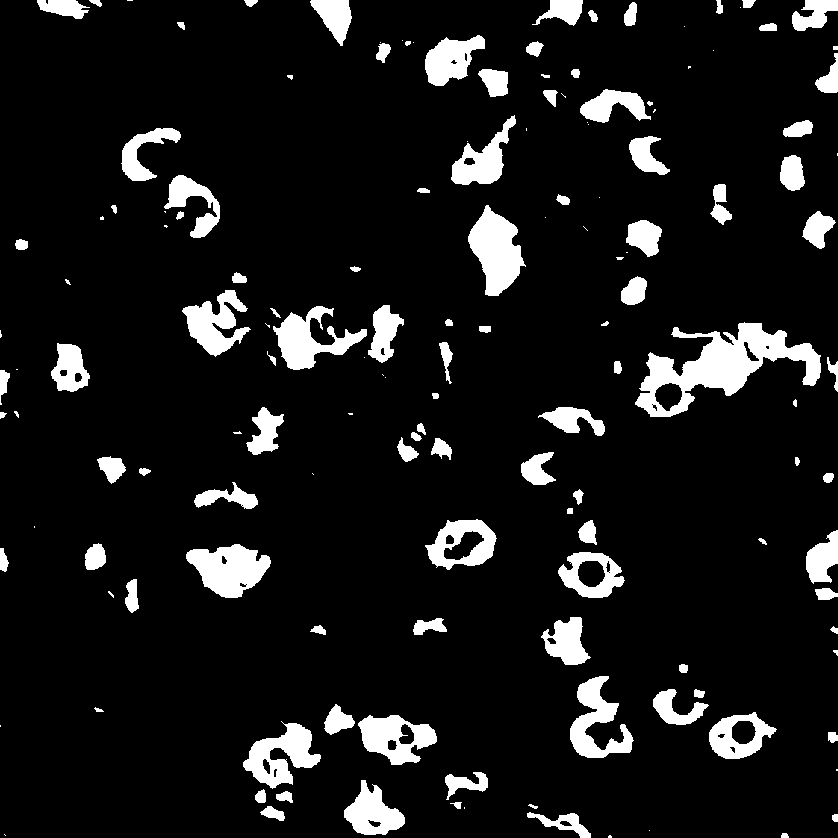

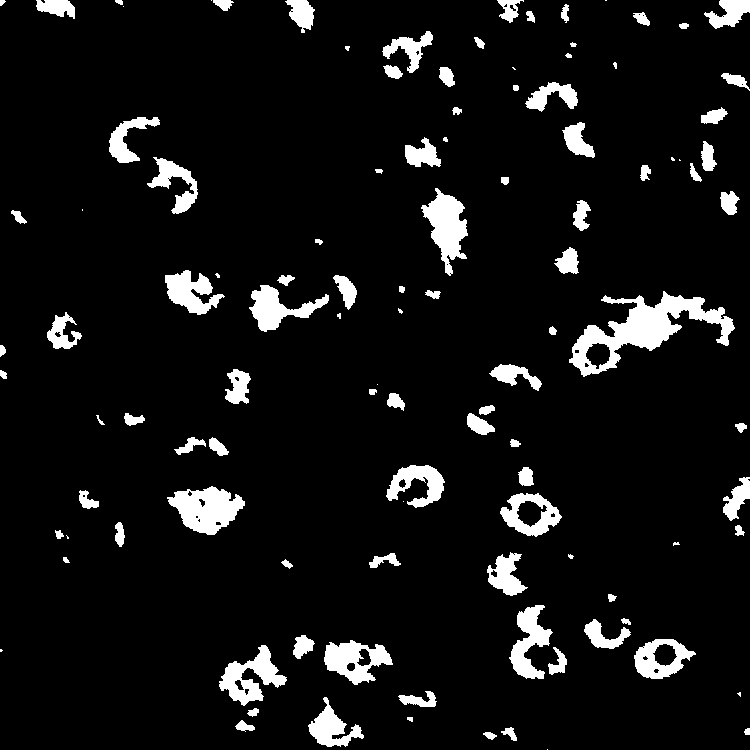

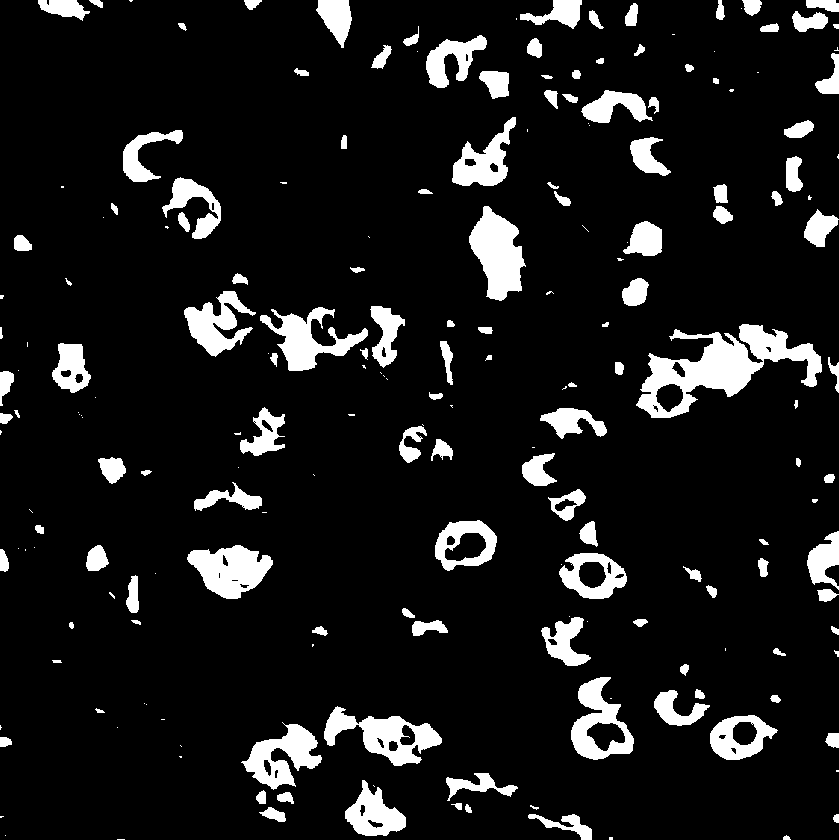

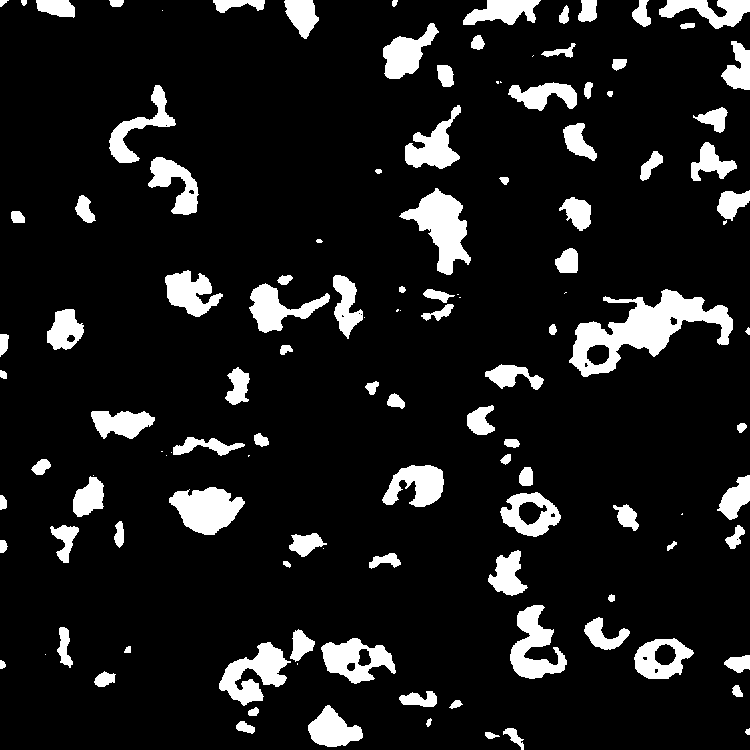

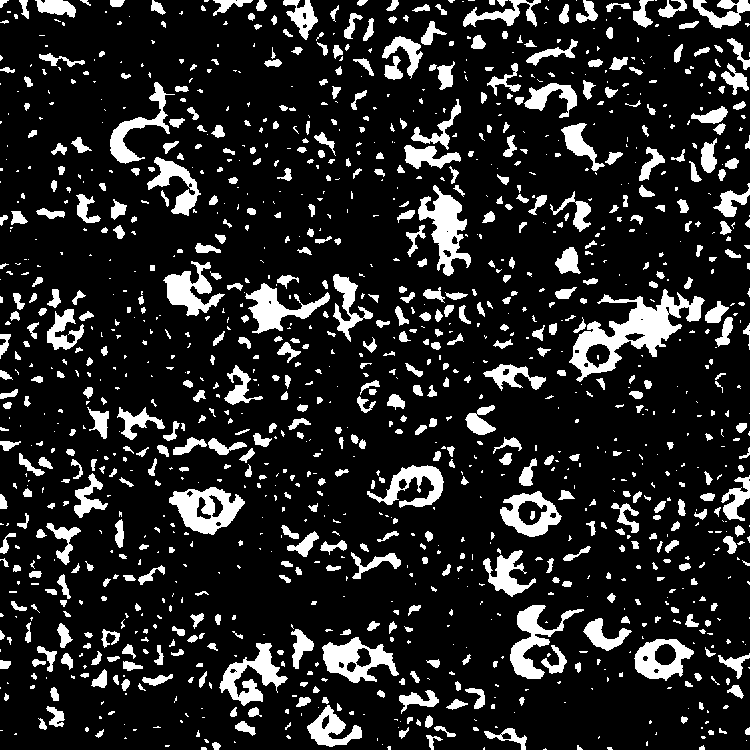


**TRTV-L_1_ (Full)**

**TRTV-L_1_**


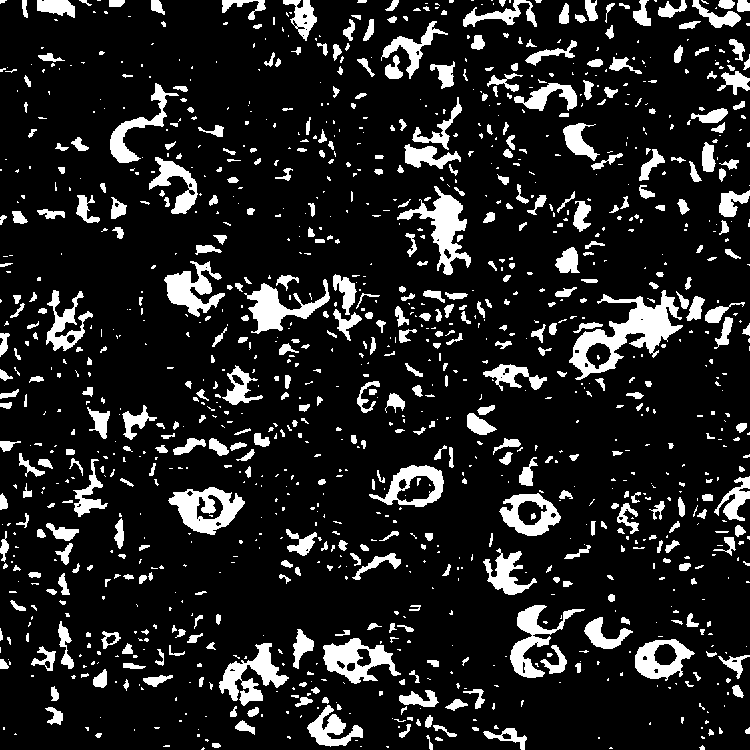


**BM3D**

**HS**

**ATRTV**

**STV**

**TRTV-L_1_ (Partial)**

**TRTV-L_1_**

S5. Segmentations of the dark holes (brain cells) within the raw image and the denoised images in Fig. 2, using manually optimized thresholds to detect most parts of the dark holes with least background included. The segmentation of the raw image indicates the strong noise present in the THG image. The segmentations of TV, EED, ATRTV, STV, TRTV-L_1_ are similar but the small objects resident in segmentations of BM3D and HS illustrate their poor denoising performance.


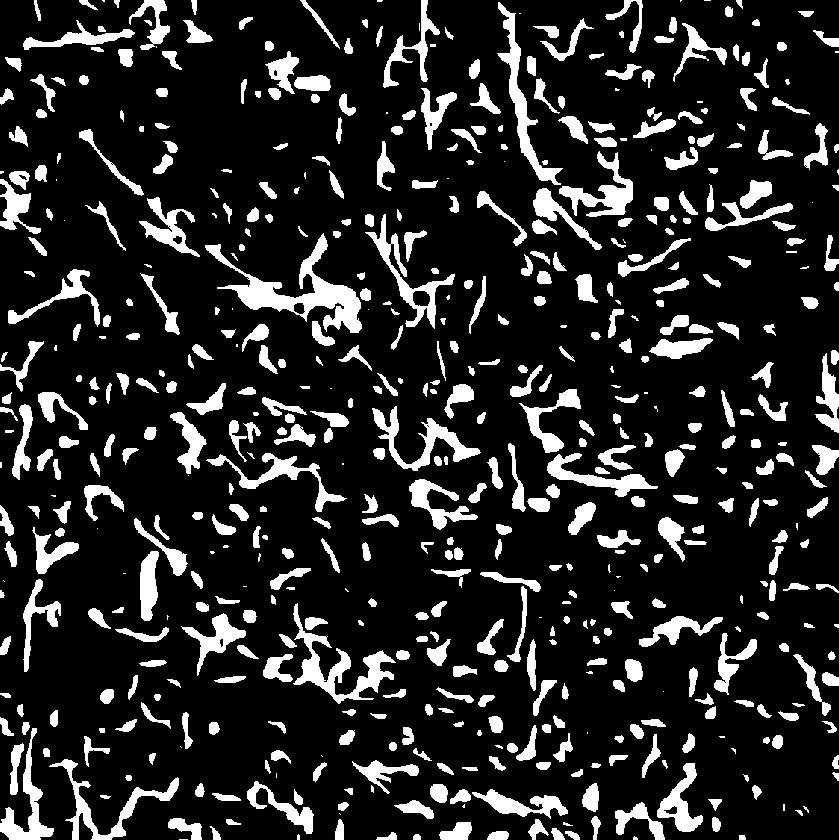

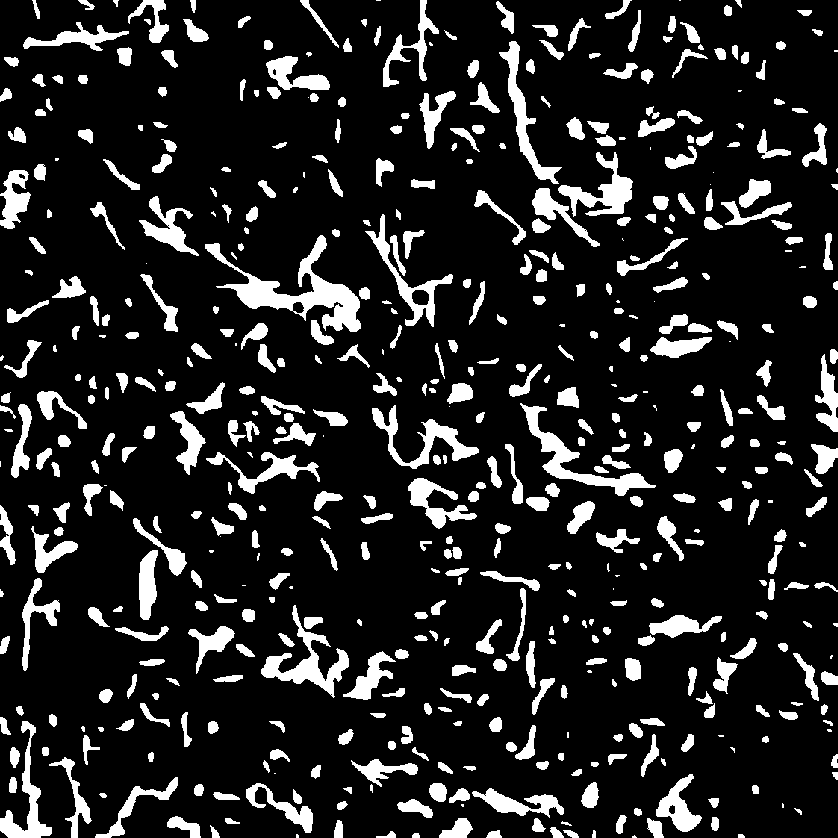

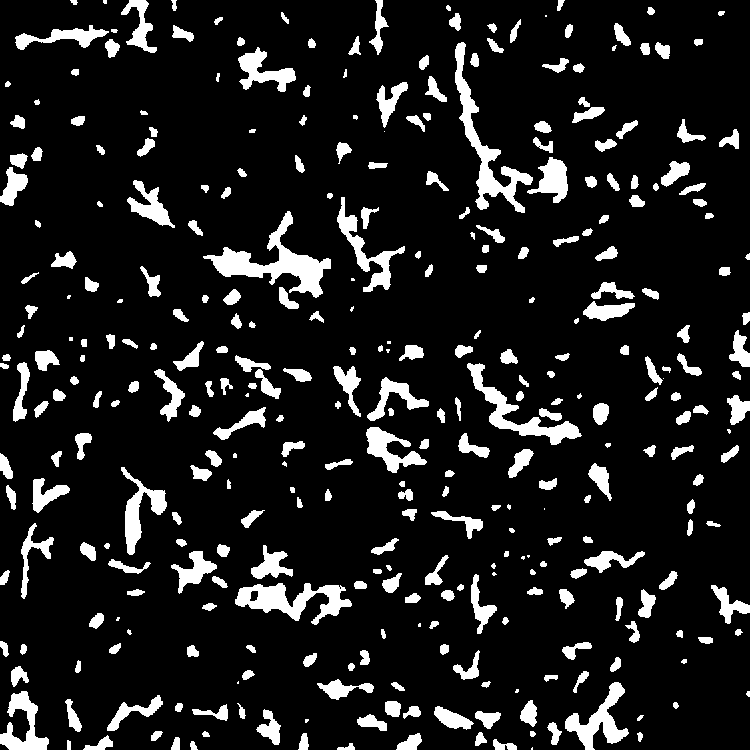

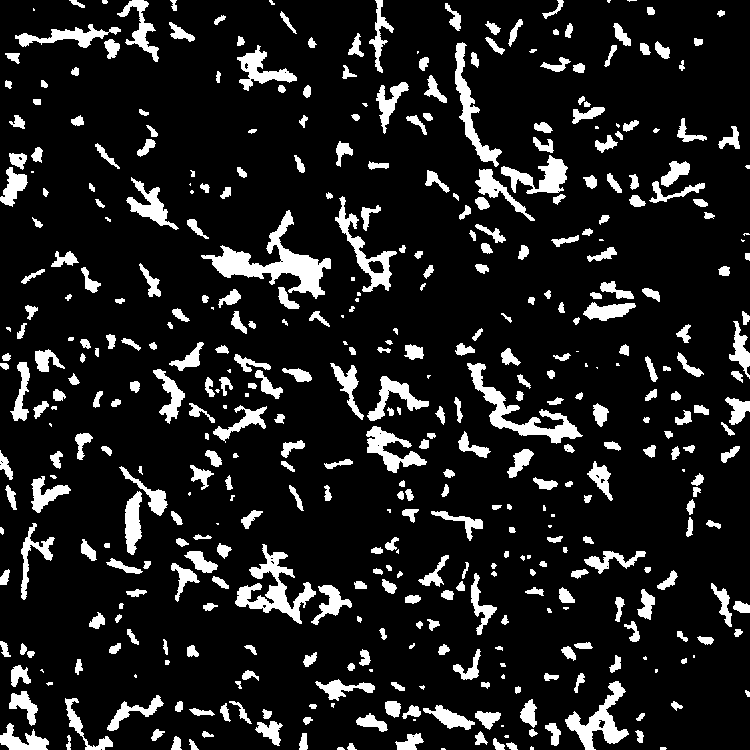

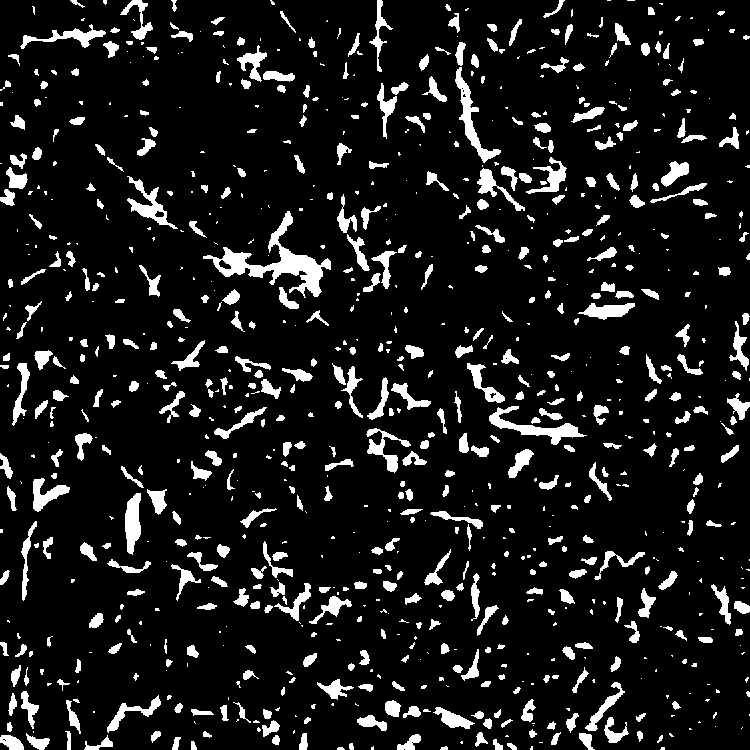

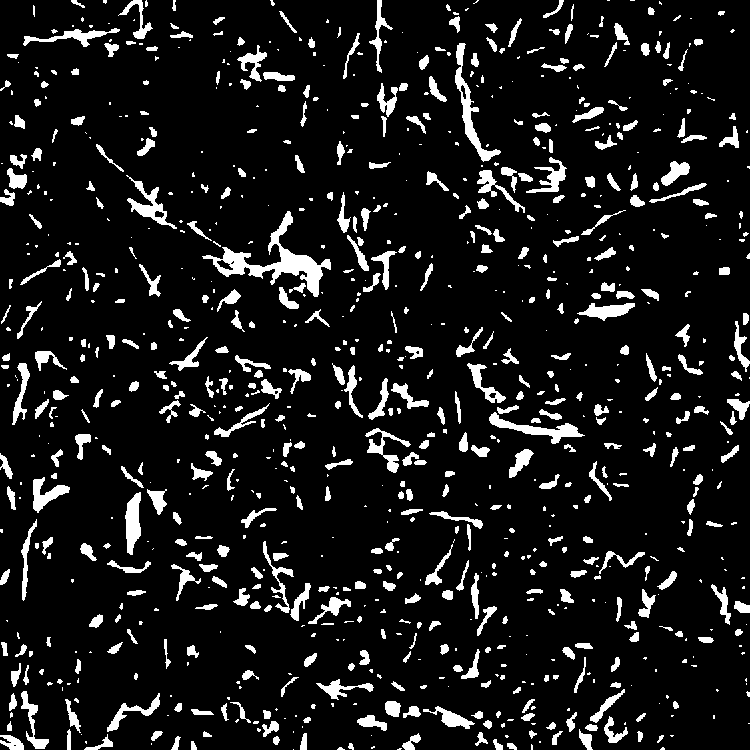

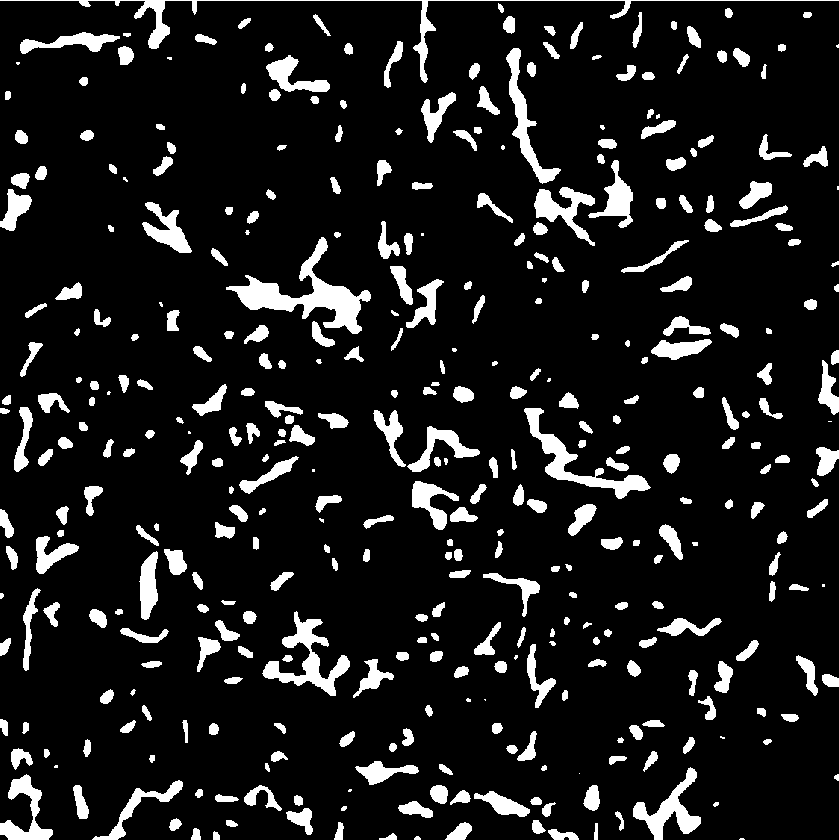

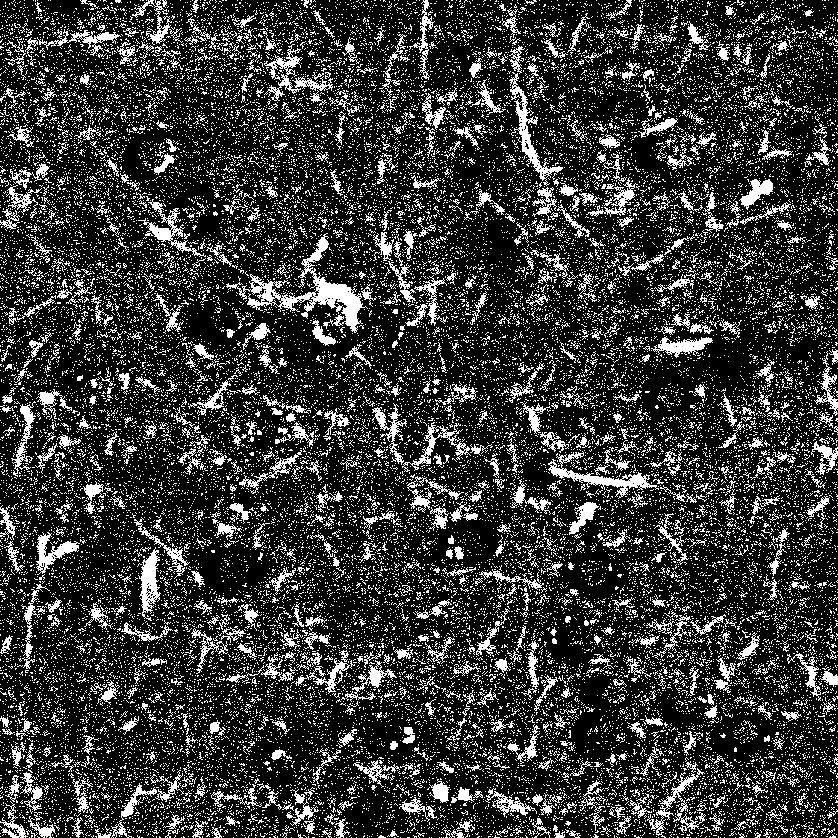

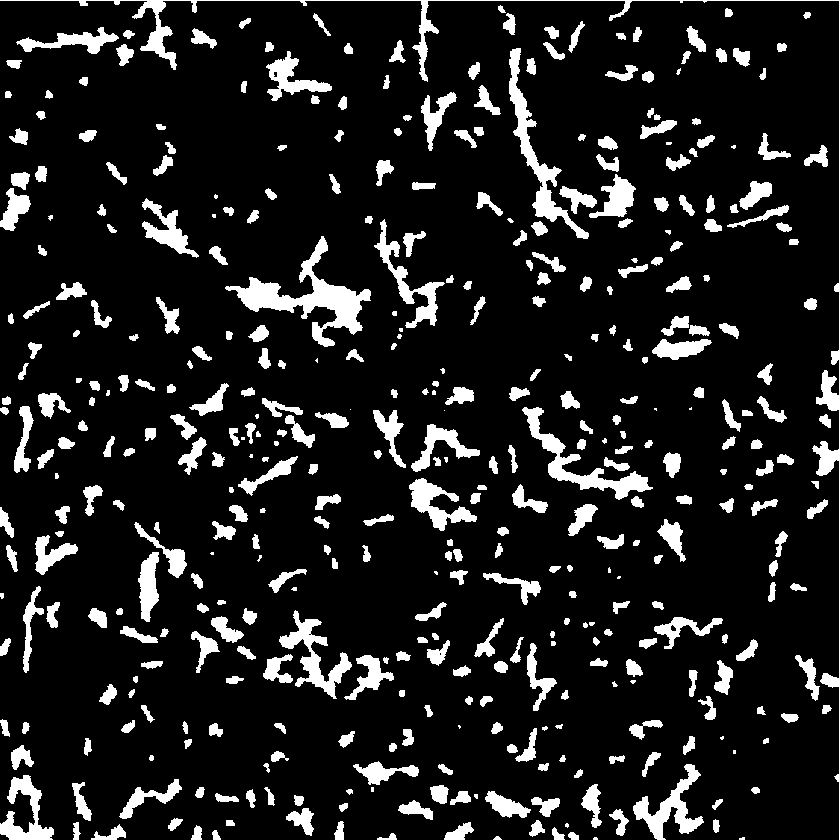


**Raw**

**TV**

**EED**

**TRTV-L_1_ (Full)**

**TRTV-L_1_**

**BM3D**

**HS**

**ATRTV**

**STV**

**TRTV-L_1_ (Partial)**

**TRTV-L_1_**

S6. Segmentations of the bright objects (neuropil) within the raw image and the denoised images in Fig. 2, using manually optimized thresholds to detect most parts of the bright objects with least background included (for example, the fiber indicated by yellow arrow). The segmentation of the raw image indicates the strong noise present. The segmentation of TRTV-L_1_ is comparable to those of BM3D and HS, where more fibers have been resolved than other models. Sometimes fibers (blue arrows) are even better segmented from the image denoised by TRTV-L_1_, which suggests that BM3D and HS could visually keep more details than TRTV-L_1_ but it is not necessarily beneficial for the segmentation followed.


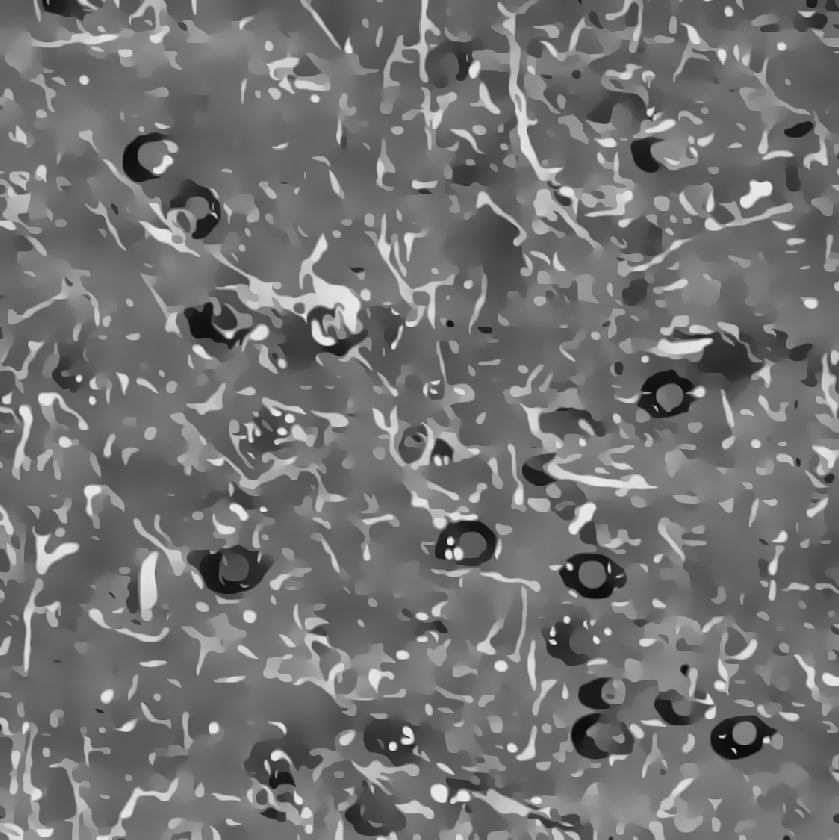

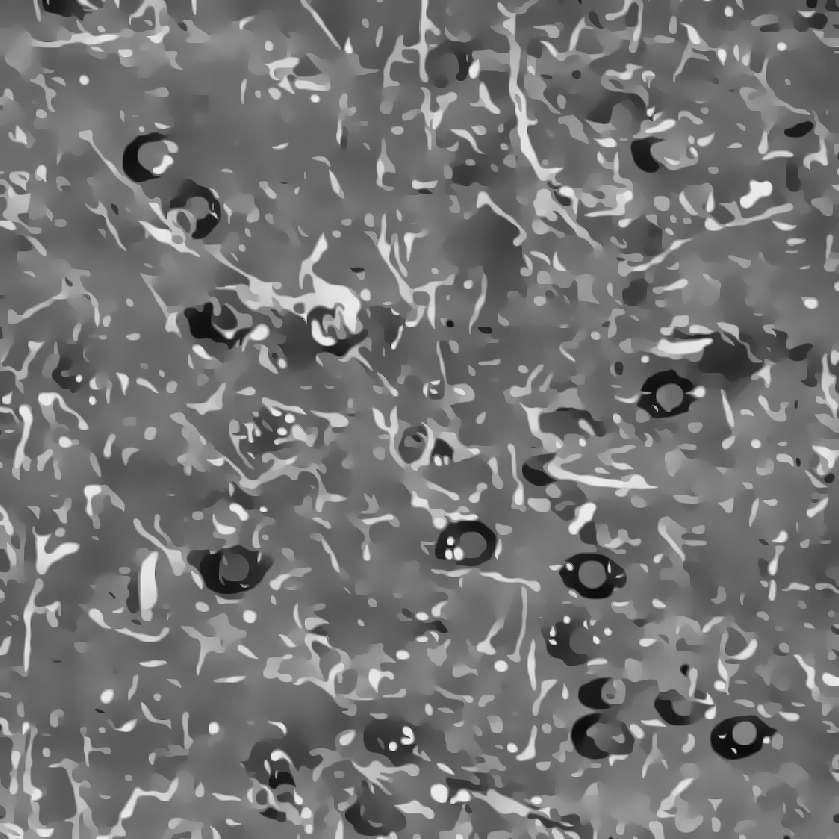

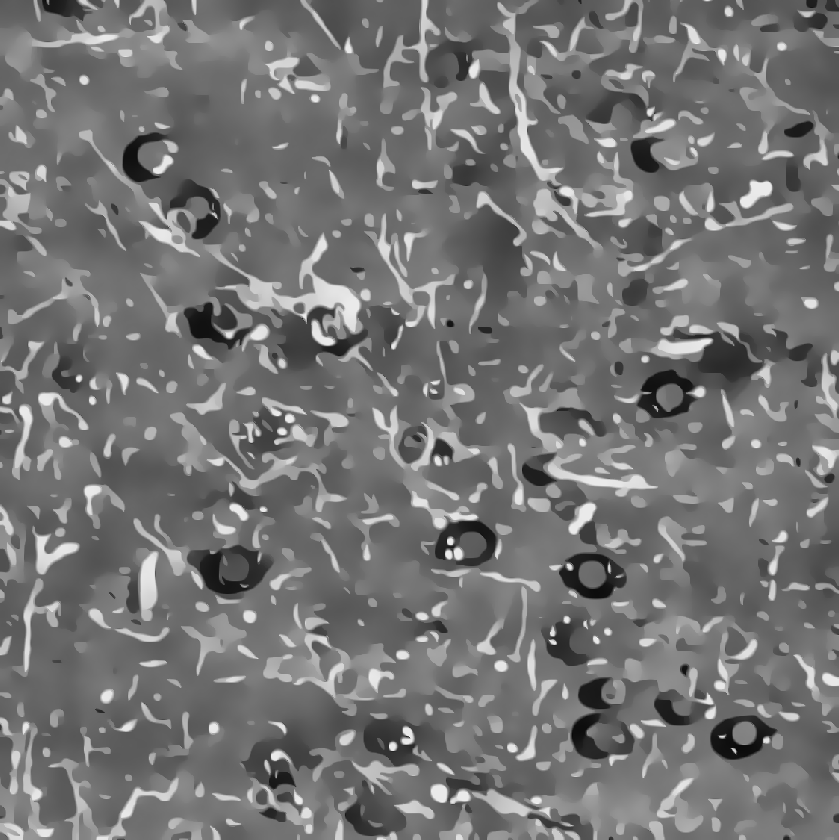

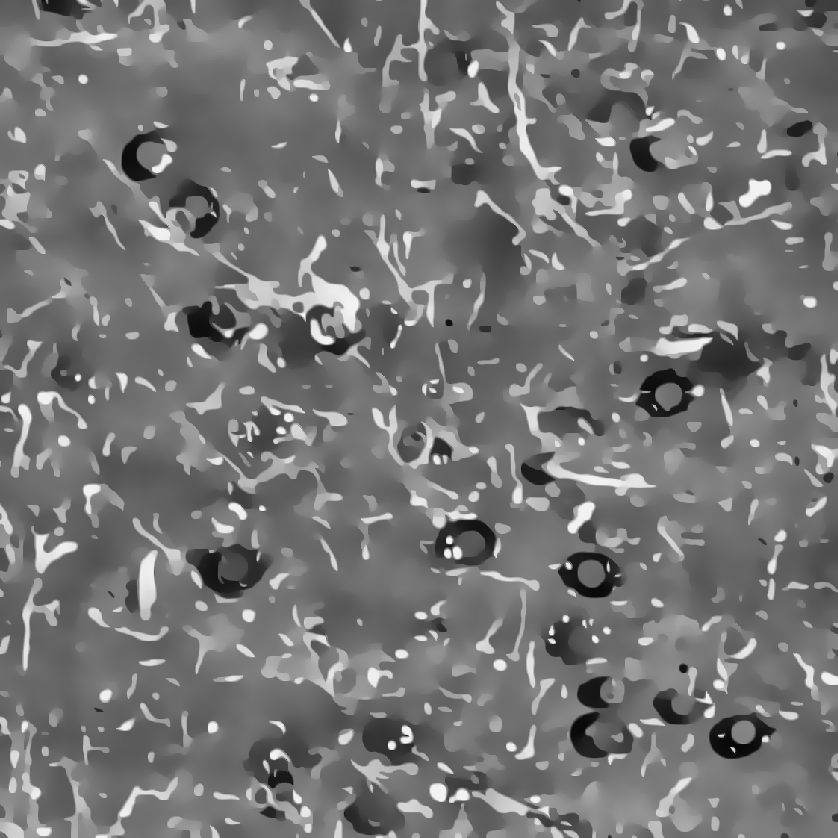

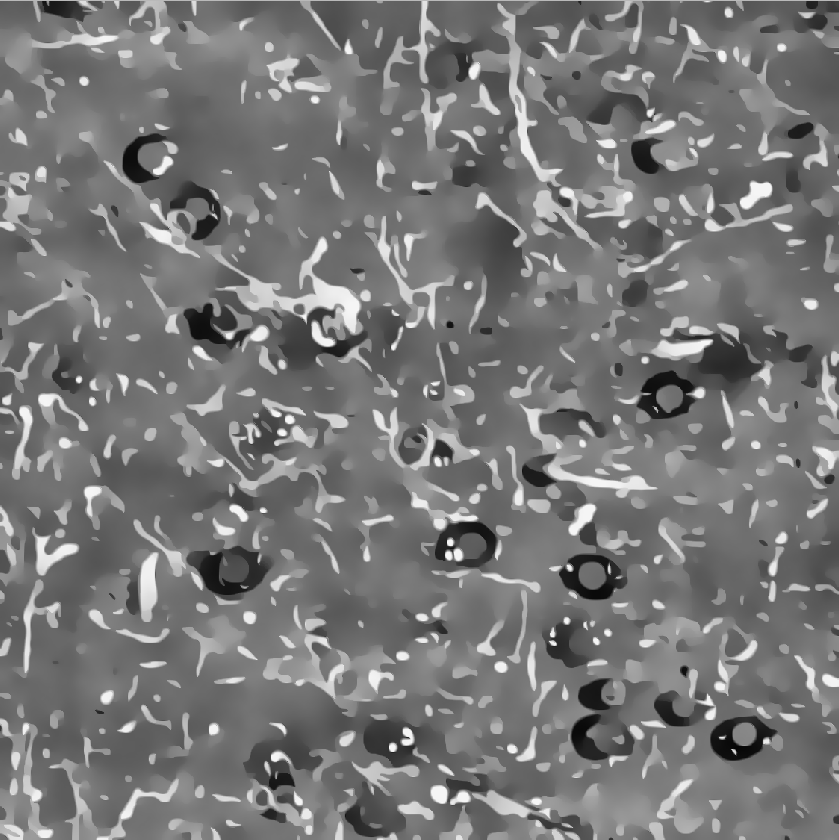

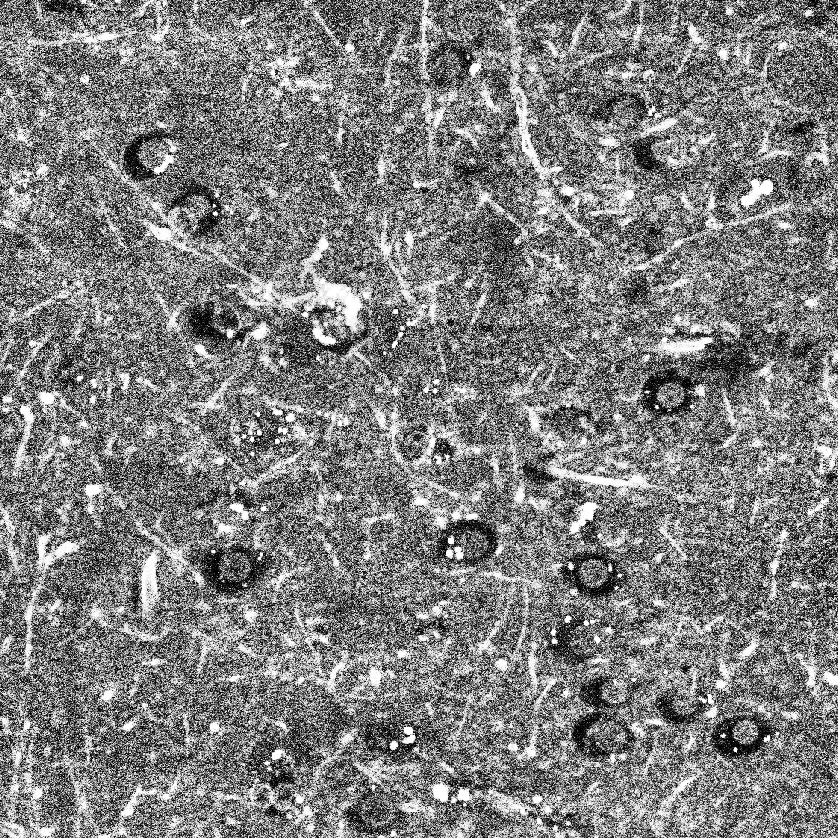


**Raw**

***h*=0.0 (Full)**

***h*=0.2**

***h*=0.8**

***h*=0.9 (Partial)**

***h*=0.5**

S7. Results of the proposed TRTV-L_1_ model for *h* = 0.0 (full estimation), 0.2, 0.5, 0.8 and 0.9 (partial estimation). Almost no degradation has been found in the restoration quality when *h* varies from 0.0 to 0.9, and thus we use *h*=0.9.
